# Supplementary material for: Scoping and targeted reviews to support development of SPIRIT and CONSORT extensions for randomised controlled trials with surrogate primary endpoints: protocol
Source: BMJ Open. 2022 Oct 13;12(10):e062798. doi: 10.1136/bmjopen-2022-062798 (PMC9562307; doi:10.1136/bmjopen-2022-062798)
Supplement: Supplementary data [file bmjopen-2022-062798supp001.pdf]

Supplementary File 1

Supplementary Table 1: Search strategy for MEDLINE

1	*Endpoint Determination/mt, st [Methods, Standards]
2	(Surrogate adj2 (measure* or outcome* or endpoint* or end point*)).ab,kw,ti.
3	(Surrogate and (controlled or trials)).ti.
4	(Endpoint adj1 determination).ab,kw,ti.
5	(Intermediate adj2 (outcome* or endpoint* or end point*)).ab,kw,ti.
6	1 or 2 or 3 or 4 or 5
7	Guideline Adherence/
8	Practice Guidelines as Topic/
9	Guidelines as Topic/
10	Checklist/
11	(Comparison or Regulation or regulatory or Policy or Decisions or Recommendation or Decision making or limitation* or understanding or reporting or critique or concept or conceptual or Validation or validity or recommendation or recommendations or guidance or advice or guideline* or guide line* or checklist or checklists or check list* or standard or standards or requirement* or instruction*).ti.
12	7 or 8 or 9 or 10 or 11
13	6 and 12
14	Clinical Trials as Topic/
15	Cohort Studies/
16	Randomized Controlled Trials as Topic/
17	"Reproducibility of Results"/
18	Research Design/
19	Data Collection/
20	Drug Approval/
21	Treatment Outcome/
22	Outcome Assessment, Health Care/
23	(outcomes or regulation).ti.
24	(clinical adj1 outcome assessment).ti.
25	clinical trials.ti.
26	14 or 15 or 16 or 17 or 18 or 19 or 20 or 21 or 22 or 23 or 24
27	13 and 26

Manyara AM, *et al. BMJ Open* 2022; 12:e062798. doi: 10.1136/bmjopen-2022-062798

Supplementary Table 2: Search strategy for EMBASE

1	(Surrogate adj2 (measure* or outcome* or endpoint* or end point*)).ab,kw,ti.
2	(Surrogate and (controlled or trials)).ti.
3	(Endpoint adj1 determination).ab,kw,ti.
4	(Intermediate adj2 (outcome* or endpoint* or end point*)).ab,kw,ti.
5	1 or 2 or 3 or 4
6	protocol compliance/
7	practice guideline/
8	checklist/
9	(Comparison or Regulation or regulatory or Policy or Decisions or Recommendation or Decision making or limitation* or understanding or reporting or critique or concept or conceptual or Validation or validity or recommendation or recommendations or guidance or advice or guideline* or guide line* or checklist or checklists or check list* or standard or standards or requirement* or instruction*).ti.
10	6 or 7 or 8 or 9
11	5 and 10
12	"clinical trial (topic)"/
13	cohort analysis/
14	"randomized controlled trial (topic)"/
15	reproducibility/
16	methodology/
17	information processing/
18	drug approval/
19	treatment outcome/
20	outcome assessment/
21	(outcomes or regulation).ti.
22	(clinical adj1 outcome assessment).ti.
23	clinical trials.ti.
24	12 or 13 or 14 or 15 or 16 or 17 or 18 or 19 or 20 or 21 or 22 or 23
25	11 and 24

Manyara AM, *et al. BMJ Open* 2022; 12:e062798. doi: 10.1136/bmjopen-2022-062798

Supplementary Table 3: Search strategy for Grey literature

Search strategy for grey literature. For each unique search, 100 hits will be reviewed for eligibility.

Source	Search strategy
**Google search engine**	Advanced search option (www.google.co.uk/advanced_search) will be used. Searches will combine terms appearing in the titles of for example:<br>• "surrogate endpoints" AND "recommendation" OR "guidance" OR "considerations"<br>• "clinical endpoints" AND "recommendation" OR "guidance" OR "considerations"
**Examples of relevant websites**	
**FDA (www.fda.gov)**	Use a search function to do a broad search using terms such as "surrogate endpoints", "clinical endpoints"
**MHRA (www.gov.uk/government/organisations/medicines-and-healthcare-products-regulatory-agency)**	No search option website will be browsed.
**European Medicines Agency (www.ema.europe.eu)**	Given its a large website, Advance Google search will be used, and search limited by the search by URL
**COMET initiative (www.comet-initiative.org)**	Searches done by options provided by the website. For example: by Method= Literature review; by Study Type =Commentary, COS methods research

Manyara AM, *et al. BMJ Open* 2022; 12:e062798. doi: 10.1136/bmjopen-2022-062798
