# Supplementary material for: Scoping and targeted reviews to support development of SPIRIT and CONSORT extensions for randomised controlled trials with surrogate primary endpoints: protocol
Source: BMJ Open. 2022 Oct 13;12(10):e062798. doi: 10.1136/bmjopen-2022-062798 (PMC9562307; doi:10.1136/bmjopen-2022-062798)
Supplement: Author's manuscript [file bmjopen-2022-062798.draft_revisions.pdf]

BMJ Open

BMJ Open is committed to open peer review. As part of this commitment we make the peer review history of every article we publish publicly available.

When an article is published we post the peer reviewers' comments and the authors' responses online. We also post the versions of the paper that were used during peer review. These are the versions that the peer review comments apply to.

The versions of the paper that follow are the versions that were submitted during the peer review process. They are not the versions of record or the final published versions. They should not be cited or distributed as the published version of this manuscript.

BMJ Open is an open access journal and the full, final, typeset and author-corrected version of record of the manuscript is available on our site with no access controls, subscription charges or pay-per-view fees (http://bmjopen.bmj.com).

If you have any questions on BMJ Open's open peer review process please email info.bmjopen@bmj.com

BMJ Open

Protocol for scoping and targeted reviews to support development of SPIRIT and CONSORT extensions for randomised controlled trials with surrogate primary endpoints

Journal:	*BMJ Open*
Manuscript ID	bmjopen-2022-062798
Article Type:	Protocol
Date Submitted by the Author:	11-Mar-2022

Complete List of Authors:	Manyara, Anthony; University of Glasgow, MRC/CSO Social and Public Health Sciences Unit, Institute of Health and Wellbeing<br>Davies, Philippa ; University of Bristol, Population Health Sciences, Bristol Medical School<br>Stewart, Derek; Patient and Public Involvement lead<br>Wells, Valerie; University of Glasgow, MRC/CSO Social and Public Health Sciences Unit, Institute of Health and Wellbeing<br>Weir, Christopher; University of Edinburgh, Edinburgh Clinical Trials Unit, Usher Institute<br>Young, Amber; University Hospitals Bristol NHS Foundation Trust, Paediatric Anaesthesia; University of Bristol, Population Health Sciences, Bristol Medical School<br>Taylor, Rod; University of Glasgow, MRC/CSO Social and Public Health Sciences Unit, Institute of Health and Wellbeing; University of Glasgow, Robertson Centre for Biostatistics, Institute of Health and Well Being<br>Ciani, Oriana ; SDA Bocconi School of Management

Keywords:	Protocols & guidelines < HEALTH SERVICES ADMINISTRATION & MANAGEMENT, EPIDEMIOLOGY, PUBLIC HEALTH

SCHOLARONE™ Manuscripts

Protocol for scoping and targeted reviews to support development of SPIRIT and CONSORT extensions for randomised controlled trials with surrogate primary endpoints

Anthony Muchai Manyara[1], Philippa Davies[2], Derek Stewart[3], Valerie Wells[1], Christopher J Weir[4], Amber Young[2], Rod S Taylor[1,4], Oriana Ciani[5]

1 MRC/CSO Social and Public Health Sciences Unit, Institute of Health and Wellbeing, University of Glasgow, UK

2 Population Health Sciences, Bristol Medical School, University of Bristol, UK

3 Patient and Public Involvement lead, UK

4 Edinburgh Clinical Trials Unit, Usher Institute, University of Edinburgh, UK

5 Robertson Centre for Biostatistics, Institute of Health and Well Being, University of Glasgow, UK

6 SDA Bocconi School of Management, Milan, Italy

Correspondence to Anthony Manyara; Anthony.Manyara@glasgow.ac.uk

MRC/CSO Social and Public Health Sciences Unit, Institute of Health and Wellbeing, University of Glasgow, UK

For peer review only

For peer review only - http://bmjopen.bmj.com/site/about/guidelines.xhtml

Abstract

Introduction

Using a surrogate endpoint as a substitute for a primary patient-relevant outcome enables randomised controlled trials (RCTs) to be conducted more efficiently, i.e., with shorter time, smaller sample size, and lower cost. However, there is currently no consensus-driven guideline for the reporting of RCTs using a surrogate endpoint as a primary outcome; therefore, we seek to develop SPIRIT (Standard Protocol Items: Recommendations for Interventional Trials) and CONSORT (Consolidated Standards of Reporting Trials) extensions to improve the design and reporting of these trials. As an initial step, scoping and targeted reviews will identify potential items for inclusion in the extensions and participants to contribute to a Delphi consensus process.

Methods and analysis

The scoping review will search and include literature reporting on the current understanding, limitations, and guidance on using surrogate endpoints in trials. Relevant literature will be identified through: 1) bibliographic databases; 2) grey literature; 3) handsearching of reference lists; and 4) solicitation from experts. Data from eligible records will be thematically analysed into potential items for inclusion in extensions. The targeted review will search for RCT reports and protocols published from 2017-2021 in six high impact general medical journals. Trial corresponding author contacts will be listed as potential participants for the Delphi exercise.

Ethics and dissemination

Ethical approval is not required. The reviews will support the development of SPIRIT and CONSORT extensions for reporting surrogate primary endpoints. The findings will be published in open-access publications.

This review has been prospectively registered in the OSF Registries Registration DOI: 10.17605/OSF.IO/WP3QH.

Keywords: Surrogate endpoints, randomised controlled trials, Reporting guidelines

For peer review only - http://bmjopen.bmj.com/site/about/guidelines.xhtml

Strengths and limitations of this study

- Our scoping review will use rigorous methods to identify literature using multiple sources with no restriction to regions or time periods.
- The targeted review will identify recent randomised controlled trials that have used surrogate primary endpoints from six high impact journals.
- Due to lack of resources for translation, we will only include records in English or Italian.
- Using a purposively selected set of journals for the targeted review means that our review of recent RCT protocols and trials is not exhaustive and may lack generalisability.

For peer review only

For peer review only - http://bmjopen.bmj.com/site/about/guidelines.xhtml

Introduction

Randomised controlled trials (RCTs), that are well designed, conducted, and reported, provide rigorous scientific evidence for evaluating the effectiveness and safety of interventions intended to impact health [1, 2]. Nevertheless, to meet the scientific, ethical, and regulatory requirements, the conduct and delivery of RCTs is becoming increasingly resource and time-intensive [3], with median cost estimates of up to US$ 21.4 million for phase three trials [4]. The use of a surrogate endpoint as a substitute for a primary final patient relevant outcome [5] provides a potentially attractive solution for improving efficiency of RCTs, i.e., shorter follow up, smaller sample size, and, as a result, lower cost.

A key rationale for the use of a surrogate endpoint is that the intervention effect on the surrogate fully captures the intervention effect on the final patient relevant outcome [6]. Consideration of surrogate endpoints in RCTs has traditionally focused on the regulatory setting for pharmaceuticals and whether biomarkers are "likely to predict" patient-centred outcomes of interest (e.g., systolic blood pressure for stroke, low density lipoprotein cholesterol for myocardial infarction, and HIV viral load for development of AIDS). However, it is important to acknowledge a more wider application in RCTs of intermediate outcomes that are believed to capture the causal pathway through which pharmaceutical, surgical, organizational or public health interventions impact the ultimate patient-relevant outcome (e.g., hospice enrolment for mortality with an intervention aimed at improving end of life care [7]; fruit and vegetable consumption for cardiovascular events for a behavioural intervention designed to improve cardiovascular risk [8]).

Despite the potential appeal of surrogate endpoints in RCTs, their use in clinical and policy decision making remains controversial. An empirical analysis has found that RCTs using a surrogate endpoint primary outcome typically report 46% larger treatment effects compared to RCTs with final patient relevant primary outcomes [9]. This finding is supported by theoretical analyses [10]. Concerningly, some approvals based on surrogate endpoints have led to the 'real world' use of interventions that fail to demonstrate their predicted benefit(s) on the ultimate patient-centred outcome of interest and even more worryingly, that result in more harm than good [11, 12]. Therefore, design and reporting of RCTs using surrogate endpoints should clearly convey the uncertainty and risks associated with their use. However, audits of RCTs to date have found this not to be the case. An analysis of 626 RCTs published in 2005 and 2006 found that 107 (17%) used a surrogate primary endpoint and of these, only

For peer review only

For peer review only - http://bmjopen.bmj.com/site/about/guidelines.xhtml

a third discussed whether the surrogate was a valid predictor of patient-relevant outcomes [13]. Furthermore, a review of 220 cardiovascular surrogate trials found that only 59 (27%) had evidence validating the benefits of interventions on a final patient-relevant outcome [14].

Reporting guidelines can guide design and improve the reporting of RCTs at both the protocol and report stages. Two established guidelines are: SPIRIT (Standard Protocol Items: Recommendations for Interventional Trials) 2013 statement: a 33-item checklist used to guide the drafting of RCT protocols [15]; and CONSORT (Consolidated Standards of Reporting Trials) 2010 Statement is a 25-item checklist used to improve reporting of conducted trials [16]. Yet, although SPIRIT and CONSORT (and related extensions) provide general guidance on outcome reporting, there remains no standard evidence-based reference for dealing with surrogacy of the primary endpoint. Improving transparency in the reporting of trials using surrogates would enable the evidence base for the surrogate to be more effectively scrutinised. Therefore, we aim to develop extensions to report trial protocols and reports that use surrogate primary endpoints: SPIRIT-SURROGATE and CONSORT-SURROGATE, respectively. Our working definition of a surrogate endpoint is: 'a biomarker or intermediate outcome used to substitute and predict for a final patient relevant outcome (i.e., characteristic or variable that captures how a patient feels, functions, or how long they survive, such as the outcomes of mortality or health-related quality of life)' [5, 6, 17].

To develop these extensions, we will closely follow the EQUATOR (Enhancing the QUAlity and Transparency Of health Research) network's recommended steps for developing a health research reporting guideline [18]. We have structured our project into four phases: Phase 1 (Literature reviews), Phase 2 (Delphi study), Phase 3 (Consensus meeting), and Phase 4 (Knowledge translation). This protocol outlines the activities and procedures of Phase 1 consisting of scoping and targeted reviews. The scoping review will be used to: synthesise current evidence and guidance on using surrogate endpoints to generate candidate items for potential inclusion in extensions; and identify surrogate content experts for recruitment in the Delphi study (Phase 2). The primary aim of the targeted review is to identify trial investigators who have led an RCT using a surrogate endpoint to be invited to participate in the Delphi exercise. A secondary aim will be to archive identified protocols and trials and use them as a 'baseline' for future evaluation of the impact of developed extensions on the reporting practice of future RCT protocols and reports.

For peer review only - http://bmjopen.bmj.com/site/about/guidelines.xhtml

Methods and analysis

Scoping review

The scoping review was considered to be the most suitable knowledge synthesis approach for addressing the broad aim of this study [19]. The scoping review will be conducted using a methodological framework proposed by Arksey and O'Malley [20], and enhancements proposed to this framework by Levac et al [21] and Peters et al [22]. This will involve six stages: formulating a research question; identifying relevant studies; inclusion of studies; charting data; summarising and reporting results; and consultation [20].

Framework stage one: Formulating the research question

This scoping review seeks to identify a list of items that should be considered when reporting RCT protocols and reports which use surrogate endpoints. Therefore, our overarching research question combines a broad scope and a specific area of inquiry [21] (i.e., surrogate endpoint use): what is the current understanding, advice, and guidance on using surrogate endpoints in RCTs? Specific research questions are:

1. How are surrogate endpoints defined?
2. What are the limitations of using surrogate endpoints in RCTs?
3. When is the use of surrogate endpoints acceptable?
4. What published advice and guidance exists on reporting RCTs protocols and reports using surrogate endpoints?

There is a possibility of modification of these research questions during the literature reviewing and this will be reported when publishing the findings.

Framework stage two: Identifying relevant literature

We will adopt a search approach that balances comprehensiveness, breadth, and feasibility [21]. Relevant literature will be identified through: 1) electronic bibliographic databases (Excerpta Medica Database (EMBASE), Medical Literature Analysis and Retrieval System Online (MEDLINE), Cochrane Methodology Register); 2) Grey literature (Google and relevant website search); 3) handsearching of reference lists; and 4) solicitation for additional literature from expert colleagues [20-22].

Electronic databases and grey literature search will be supported by an experienced information specialist (VW). We have developed an initial search strategy for MEDLINE and EMBASE which combines “surrogate endpoints”, “guidelines”, and “trials” related search

For peer review only - http://bmjopen.bmj.com/site/about/guidelines.xhtml

terms (see Supplementary Table 1 and Supplementary Table 2 in the Supplementary File). This strategy was checked for validity against four highly cited articles (>50 citations) that answer either of our specific research questions [9, 13, 23, 24].

For grey literature, search strategies will be modified for each of the websites and for each strategy, the search terms and the number of results retrieved and/or screened will be recorded [25]. Supplementary Table 3 shows search strategies to be used in the Google search engine and in some of the relevant websites. Generally, the strategies will include combination of search terms (e.g., "surrogate endpoints" AND "guidance") in Google advanced search; broad searches (e.g., surrogate endpoints) using the website search function; and browsing for websites without a search function. For large websites (e.g., www.ema.europe.eu), Google advanced search will be used, and search limited to the website URL. The first 100 hits in each search will be screened for eligibility to balance between feasibility and relevancy of records [25]. One reviewer will screen searches on the Google search engine or websites using title and, if present, any short text underneath.

All reference lists of included full texts will be screened to identify relevant records. We will solicit for additional resources from surrogate and outcome measurement experts including authors of a recent scoping review (on "outcome reporting recommendations for trial protocols and reports") which identified eight documents that focused on reporting recommendations for surrogate outcomes [26].

Framework stage three: Literature selection

Databases search results will be exported to Endnote version X9 for the removal of duplicates. The remaining records will be exported to Covidence [27] for eligibility screening based on title, abstract, and full-text reading by two reviewers. Title and abstract screening of grey literature will be done in respective websites by one reviewer and full-text screening done from HTML files by two reviewers.

Once full-text screening has been concluded, reviewers will hand search reference lists of all included full-texts for relevant records. The identified records combined with those supplied from experts will undergo full-text screening. Records will be eligible for inclusion if they report findings relevant to any research question. While we will mainly include records that are peer-reviewed literature, academic or regulatory grey literature (e.g., white papers), reviewers will make judgements on inclusion of other records (e.g., conference abstracts) based on relevance to review questions and trustworthiness of evidence presented. We will

For peer review only - http://bmjopen.bmj.com/site/about/guidelines.xhtml

not restrict our inclusion of literature to regions or time periods. However, we will only include records in English or Italian due to lack of resources for translation. Disagreements between reviewers will be resolved by consensus or, if necessary, involving a third reviewer.

Framework stage four: Charting the findings

The following data will be extracted: author (and contact of corresponding author), publication year, country, author affiliation category (e.g., academic, regulatory body, patient/public forums), record type (e.g., review article, commentary, regulatory guidance), research area if specified, funding if stated, and findings relevant to research questions (i.e., definition, limitations, acceptability, guidance on surrogate endpoints use). A pilot will be undertaken to check if the data extraction template needs modification. All data extraction will be done by one reviewer. At the start of extraction, a subset of extracted data (~10% of records) will be checked for accuracy by a second reviewer and if accurate the first reviewer will proceed to extract in all other records.

Framework stage five: Synthesis and reporting the findings

All analysis will be done in Microsoft Excel. Descriptive data (i.e., publication year, country/region, author affiliation category, record type) will be analysed using counts and percentages and presented in tables, graphs or as text. Data related to research questions (e.g., key messages/advice/guidelines on surrogate endpoints use) will be collated verbatim under each research question. A simple form of thematic analysis [28] will then be used to synthesise data. Two reviewers will independently read the collated data under each research question and for each record, summarise it into: 1) item(s) to be considered when reporting protocols and trials using surrogate endpoints; and 2) whether the items are new or modifications to the SPIRIT and/or CONSORT checklist items and for new items, the section of the checklist where they should be reported. The reviewers will then meet for a virtual workshop to discuss and agree on items and their designated sections of the checklist. We will report the findings in open-access peer reviewed publication using the Preferred Reporting Items for Systematic reviews and Meta-Analysis for Scoping Reviews (PRISMA-ScR) [29].

Framework stage six: Consultation exercise

The aim of consultation is to share scoping review findings with stakeholders so as to identify additional relevant resources and valuable insights that the scoping review findings may have missed [20]. Nevertheless, it is important to specify when, how, and why to do consultation,

For peer review only - http://bmjopen.bmj.com/site/about/guidelines.xhtml

the types of stakeholder involved, and how to integrate the information with review findings [21]. We will use preliminary review findings to seek insights, through virtual meetings, from Public and Patient Involvement (PPI) representatives on the identified items for reporting surrogate endpoints. Our project PPI Lead (DS) will coordinate consultation with PPI representatives, and this will offer an opportunity for knowledge transfer and exchange. Additionally, we will invite our multidisciplinary expert advisory Executive Committee members (see Acknowledgement) and the MRC-NIHR Trials Methodology Research Partnership Outcomes Working Group (www.methodologyhubs.mrc.ac.uk/), specifically the Surrogate Outcomes subgroup, to comment on any additional resources, items, and perspectives not included in the preliminary findings. Review comments on the preliminary findings document or detailed notes taken during consultation meetings will be used in summarising and integrating suggested items into the review findings.

Targeted review

The targeted reviews are intended to identify trial investigators who have led an RCT assessing a surrogate endpoint and protocols and trials that have a primary surrogate endpoint [9]. MEDLINE through PubMed will be searched for RCTs published in the last five years (2017-2021) in six high impact general medical journals: *Annals of Internal Medicine*, *BMJ*, *Journal of the American Medical Association*, *New England Journal of Medicine*, *Lancet*, and *PLoS Medicine.* Use of general medical journals allows for inclusion of records across a range of clinical areas. Given the focus of the project on reporting guidelines for trial protocols, we also will search two journals widely used for publishing RCT protocols: *BMJ Open* and *Trials*. We will include trial protocols and reports that use outcomes that meet our working definition of surrogate endpoints.

All identified protocols and trials will be exported to Endnote version X9 for the removal of duplicates and exported to Covidence [27] for eligibility screening. Given the primary objective of this review is to identify trial investigators who have used surrogate endpoints, screening will be limited to titles and abstracts. Two reviewers will screen all records and include those protocols and randomised trials that use surrogate primary endpoints and report intervention studies. A more in-depth screening and analysis of the full texts will be done as part of an upcoming project, acting as a baseline to evaluate the impact of the extensions (post-publication) on the reporting of RCT protocols and trials.

For peer review only - http://bmjopen.bmj.com/site/about/guidelines.xhtml

From the included records, one reviewer will extract the title, journal, year of publication, research area, corresponding author name, institutional affiliation, and email address. These data will be used to sample and recruit participants for the Delphi study (Phase 2 of the project).

Patient and public involvement

One of the project team members (DS) is a leading PPI advocate who has been involved in health research at local, national, and international level. As outlined, PPI will be integrated in stage six of the scoping review. We are additionally exploring how patients and the public can be meaningfully involved in this project.

Limitations

Although we will use four strategies in our scoping review searches, our inclusion will be limited to records in English and Italian language hence exclusion of non-English/Italian literature. Nevertheless, our review does not aim to be exhaustive but to identify important items for consideration when using surrogate endpoints and it is highly likely items synthesised from records in the English and Italian language would be transferable to other settings. Using an approach of a purposively selected set of journals means our targeted review of recent RCT protocols and trials is not exhaustive and may lack generalisability.

Conclusion

This protocol has described the procedures to be followed in conducting a scoping review and targeted review to support development of SPIRIT and CONSORT extensions of RCTs reporting primary outcomes that are surrogate endpoints. Use of scoping review methodology to identify candidate items to be rated by experts through a Delphi methodology [30] is consistent with EQUATOR guidelines [18] and has been used in recent developed extensions including CONSORT-ROUTINE [31] and Adaptive designs CONSORT Extension (ACE) [32]. Our targeted review will provide a 'baseline' of current RCT reporting that can be used to assess the impact of our developed extensions on future RCTs. The SPIRIT-SURROGATE and CONSORT-SURROGATE extensions seek to improve transparency of reporting and design of RCTs that use surrogate endpoints and thereby contribute to better clinical and policy decision-making, and ultimately health of the population.

For peer review only - http://bmjopen.bmj.com/site/about/guidelines.xhtml

Ethics and dissemination

The reviews do not require ethics approval. The reviews findings will be disseminated through conference presentations and open-access publications.

Contributorship Statement

PD, CJW, AY, RT, and OC were involved in funding acquisition. AMM, PD, DS, CJW, AY, RT, and OC were involved in the initial phases of study conception and design. AMM, VW, RT, and OC were involved in design of the search strategy and responsible for the first draft of the manuscript. PD, DS, CJW, and AY reviewed the first draft and all authors approved the final version.

Competing Interest

None to declare.

Funding

The development of SPIRIT and CONSORT extensions has been funded by the Medical Research Council (PD, CJW, AY, RT, OC, grant number MR/V038400/1).

Data Sharing Statement

Data collated in this study will be shared with the review findings open-access publication.

Acknowledgments

We acknowledge our advisory Executive Committee members who will oversight the project and contribute to the consultation stage of the scoping review: Professor Joseph Ross (Chair of the Executive Committee); Professor Martin Offringa; Dr. Nancy Butcher; Professor An-Wen Chan; Professor Gary Collins; Professor Sylwia Bujkiewicz; Dr Dalia Dawoud; and Dr Mario Ouwens.

References

1. Akobeng, A.K., *Understanding randomised controlled trials.* Archives of Disease in Childhood, 2005. **90**(8): p. 840-844.
2. Hariton, E. and J.J. Locascio, *Randomised controlled trials - the gold standard for effectiveness research: Study design: randomised controlled trials.* BJOG : an international journal of obstetrics and gynaecology, 2018. **125**(13): p. 1716-1716.
3. Umscheid, C.A., D.J. Margolis, and C.E. Grossman, *Key concepts of clinical trials: a narrative review.* Postgraduate medicine, 2011. **123**(5): p. 194-204.

For peer review only - http://bmjopen.bmj.com/site/about/guidelines.xhtml

4. Martin, L., et al., *How much do clinical trials cost?* Nature Reviews Drug Discovery, 2017. **16**(6): p. 381-382.
5. Biomarkers Definitions Working Group, *Biomarkers and surrogate endpoints: Preferred definitions and conceptual framework.* Clinical Pharmacology & Therapeutics, 2001. **69**(3): p. 89-95.
6. DeMets, D.L., B.M. Psaty, and T.R. Fleming, *When Can Intermediate Outcomes Be Used as Surrogate Outcomes?* Jama, 2020. **323**(12): p. 1184-1185.
7. Casarett, D., et al., *Improving the use of hospice services in nursing homes: a randomized controlled trial.* Jama, 2005. **294**(2): p. 211-7.
8. Domke, A., et al., *Immediate effects of a very brief planning intervention on fruit and vegetable consumption: A randomized controlled trial.* Applied Psychology: Health and Well-Being, 2021. **13**(2): p. 377-393.
9. Ciani, O., et al., *Comparison of treatment effect sizes associated with surrogate and final patient relevant outcomes in randomised controlled trials: meta-epidemiological study.* BMJ : British Medical Journal, 2013. **346**: p. f457.
10. Walter, S.D., et al., *Treatment effects on patient-important outcomes can be small, even with large effects on surrogate markers.* J Clin Epidemiol, 2012. **65**(9): p. 940-5.
11. Grimes, D.A. and K.F. Schulz, *Surrogate end points in clinical research: hazardous to your health.* Obstet Gynecol, 2005. **105**(5 Pt 1): p. 1114-8.
12. Thomas R. Fleming and David L. DeMets, *Surrogate End Points in Clinical Trials: Are We Being Misled?* Annals of Internal Medicine, 1996. **125**(7): p. 605-613.
13. la Cour, J.L., J. Brok, and P.C. Gøtzsche, *Inconsistent reporting of surrogate outcomes in randomised clinical trials: cohort study.* BMJ (Clinical research ed.), 2010. **341**: p. c3653-c3653.
14. Bikdeli, B., et al., *Two Decades of Cardiovascular Trials With Primary Surrogate Endpoints: 1990-2011.* Journal of the American Heart Association, 2017. **6**(3): p. e005285.
15. Chan, A.W., et al., *SPIRIT 2013 statement: defining standard protocol items for clinical trials.* Ann Intern Med, 2013. **158**(3): p. 200-7.
16. Schulz, K.F., et al., *CONSORT 2010 Statement: updated guidelines for reporting parallel group randomised trials.* BMC Medicine, 2010. **8**(1): p. 18.
17. Ciani, O., et al., *Validity of Surrogate Endpoints and Their Impact on Coverage Recommendations: A Retrospective Analysis across International Health Technology Assessment Agencies.* Med Decis Making, 2021. **41**(4): p. 439-452.
18. Moher, D., et al., *Guidance for developers of health research reporting guidelines.* PLoS Med, 2010. **7**(2): p. e1000217.
19. Munn, Z., et al., *Systematic review or scoping review? Guidance for authors when choosing between a systematic or scoping review approach.* BMC Medical Research Methodology, 2018. **18**(1): p. 143.
20. Arksey, H. and L. O'Malley, *Scoping studies: towards a methodological framework.* International Journal of Social Research Methodology, 2005. **8**(1): p. 19-32.
21. Levac, D., H. Colquhoun, and K.K. O'Brien, *Scoping studies: advancing the methodology.* Implementation Science, 2010. **5**(1): p. 69.
22. Peters, M.D.J., et al., *Guidance for conducting systematic scoping reviews.* JBI Evidence Implementation, 2015. **13**(3).
23. Fleming, T.R. and D.L. DeMets, *Surrogate end points in clinical trials: are we being misled?* Ann Intern Med, 1996. **125**(7): p. 605-13.
24. Kemp, R. and V. Prasad, *Surrogate endpoints in oncology: when are they acceptable for regulatory and clinical decisions, and are they currently overused?* BMC medicine, 2017. **15**(1): p. 1-7.
25. Godin, K., et al., *Applying systematic review search methods to the grey literature: a case study examining guidelines for school-based breakfast programs in Canada.* Systematic reviews, 2015. **4**: p. 138-138.
26. Butcher, N.J., et al., *Outcome reporting recommendations for clinical trial protocols and reports: a scoping review.* Trials, 2020. **21**(1): p. 620.

For peer review only - http://bmjopen.bmj.com/site/about/guidelines.xhtml

27. Covidence, *Covidence systematic review software, Veritas Health Innovation, Melbourne, Australia.*
28. Braun, V. and V. Clarke, *Using thematic analysis in psychology.* Qualitative research in psychology, 2006. **3**(2): p. 77-101.
29. McGowan, J., et al., *Reporting scoping reviews-PRISMA ScR extension.* J Clin Epidemiol, 2020. **123**: p. 177-179.
30. Trevelyan, E.G. and N. Robinson, *Delphi methodology in health research: how to do it?* European Journal of Integrative Medicine, 2015. **7**(4): p. 423-428.
31. Kwakkenbos, L., et al., *Protocol for a scoping review to support development of a CONSORT extension for randomised controlled trials using cohorts and routinely collected health data.* BMJ Open, 2018. **8**(8): p. e025266.
32. Dimairo, M., et al., *Development process of a consensus-driven CONSORT extension for randomised trials using an adaptive design.* BMC Medicine, 2018. **16**(1): p. 210.

For peer review only

For peer review only - http://bmjopen.bmj.com/site/about/guidelines.xhtml

Supplementary File

Supplementary Table 1: Search strategy for MEDLINE

1	*Endpoint Determination/mt, st [Methods, Standards]
2	(Surrogate adj2 (measure* or outcome* or endpoint* or end point*)).ab,kw,ti.
3	(Surrogate and (controlled or trials)).ti.
4	(Endpoint adj1 determination).ab,kw,ti.
5	(Intermediate adj2 (outcome* or endpoint* or end point*)).ab,kw,ti.
6	1 or 2 or 3 or 4 or 5
7	Guideline Adherence/
8	Practice Guidelines as Topic/
9	Guidelines as Topic/
10	Checklist/
11	(Comparison or Regulation or regulatory or Policy or Decisions or Recommendation or Decision making or limitation* or understanding or reporting or critique or concept or conceptual or Validation or validity or recommendation or recommendations or guidance or advice or guideline* or guide line* or checklist or checklists or check list* or standard or standards or requirement* or instruction*).ti.
12	7 or 8 or 9 or 10 or 11
13	6 and 12
14	Clinical Trials as Topic/
15	Cohort Studies/
16	Randomized Controlled Trials as Topic/
17	"Reproducibility of Results"/
18	Research Design/
19	Data Collection/
20	Drug Approval/
21	Treatment Outcome/
22	Outcome Assessment, Health Care/
23	(outcomes or regulation).ti.
24	(clinical adj1 outcome assessment).ti.
25	clinical trials.ti.
26	14 or 15 or 16 or 17 or 18 or 19 or 20 or 21 or 22 or 23 or 24
27	13 and 26

For peer review only - http://bmjopen.bmj.com/site/about/guidelines.xhtml

Supplementary Table 2: Search strategy for EMBASE

1	(Surrogate adj2 (measure* or outcome* or endpoint* or end point*)).ab,kw,ti.
2	(Surrogate and (controlled or trials)).ti.
3	(Endpoint adj1 determination).ab,kw,ti.
4	(Intermediate adj2 (outcome* or endpoint* or end point*)).ab,kw,ti.
5	1 or 2 or 3 or 4
6	protocol compliance/
7	practice guideline/
8	checklist/
9	(Comparison or Regulation or regulatory or Policy or Decisions or Recommendation or Decision making or limitation* or understanding or reporting or critique or concept or conceptual or Validation or validity or recommendation or recommendations or guidance or advice or guideline* or guide line* or checklist or checklists or check list* or standard or standards or requirement* or instruction*).ti.
10	6 or 7 or 8 or 9
11	5 and 10
12	"clinical trial (topic)"/
13	cohort analysis/
14	"randomized controlled trial (topic)"/
15	reproducibility/
16	methodology/
17	information processing/
18	drug approval/
19	treatment outcome/
20	outcome assessment/
21	(outcomes or regulation).ti.
22	(clinical adj1 outcome assessment).ti.
23	clinical trials.ti.
24	12 or 13 or 14 or 15 or 16 or 17 or 18 or 19 or 20 or 21 or 22 or 23
25	11 and 24

For peer review only

For peer review only - http://bmjopen.bmj.com/site/about/guidelines.xhtml

Supplementary Table 3: Search strategy for Grey literature

Search strategy for grey literature. For each unique search, 100 hits will be reviewed for eligibility.

Source	Search strategy
**Google search engine**	Advanced search option (www.google.co.uk/advanced_search) will be used. Searches will combine terms appearing in the titles of for example: • "surrogate endpoints" AND "recommendation" OR "guidance" OR "considerations" • "clinical endpoints" AND "recommendation" OR "guidance" OR "considerations"
**Examples of relevant websites**	
**FDA (www.fda.gov)**	Use a search function to do a broad search using terms such as "surrogate endpoints", "clinical endpoints"
**MHRA (www.gov.uk/government/organisations/medicines-and-healthcare-products-regulatory-agency)**	No search option website will be browsed.
**European Medicines Agency (www.ema.europe.eu)**	Given its a large website, Advance Google search will be used, and search limited by the search by URL
**COMET initiative (www.comet-initiative.org)**	Searches done by options provided by the website. For example: by Method= Literature review; by Study Type =Commentary, COS methods research

For peer review only

For peer review only - http://bmjopen.bmj.com/site/about/guidelines.xhtml

BMJ Open

Protocol for scoping and targeted reviews to support development of SPIRIT and CONSORT extensions for randomised controlled trials with surrogate primary endpoints

Journal:	*BMJ Open*
Manuscript ID	bmjopen-2022-062798.R1
Article Type:	Protocol
Date Submitted by the Author:	21-Jul-2022
Complete List of Authors:	Manyara, Anthony; University of Glasgow, MRC/CSO Social and Public Health Sciences Unit, Institute of Health and Wellbeing<br>Davies, Philippa ; University of Bristol, Population Health Sciences, Bristol Medical School<br>Stewart, Derek; Patient and Public Involvement lead<br>Wells, Valerie; University of Glasgow, MRC/CSO Social and Public Health Sciences Unit, Institute of Health and Wellbeing<br>Weir, Christopher; University of Edinburgh, Edinburgh Clinical Trials Unit, Usher Institute<br>Young, Amber; University Hospitals Bristol NHS Foundation Trust, Paediatric Anaesthesia; University of Bristol, Population Health Sciences, Bristol Medical School<br>Taylor, Rod; University of Glasgow, MRC/CSO Social and Public Health Sciences Unit, Institute of Health and Wellbeing; University of Glasgow, Robertson Centre for Biostatistics, Institute of Health and Well Being<br>Ciani, Oriana ; SDA Bocconi School of Management
**Primary Subject Heading**:	Research methods
Secondary Subject Heading:	Epidemiology, Patient-centred medicine, Medical publishing and peer review
Keywords:	Protocols & guidelines < HEALTH SERVICES ADMINISTRATION & MANAGEMENT, EPIDEMIOLOGY, PUBLIC HEALTH

SCHOLARONE™
Manuscripts

Protocol for scoping and targeted reviews to support development of SPIRIT and CONSORT extensions for randomised controlled trials with surrogate primary endpoints

Anthony Muchai Manyara[1], Philippa Davies[2], Derek Stewart[3], Valerie Wells[1], Christopher J Weir[4], Amber Young[2], Rod S Taylor[1,4], Oriana Ciani[5]

1 MRC/CSO Social and Public Health Sciences Unit, Institute of Health and Wellbeing, University of Glasgow, UK

2 Population Health Sciences, Bristol Medical School, University of Bristol, UK

3 Patient and Public Involvement lead, UK

4 Edinburgh Clinical Trials Unit, Usher Institute, University of Edinburgh, UK

5 Robertson Centre for Biostatistics, Institute of Health and Well Being, University of Glasgow, UK

6 SDA Bocconi School of Management, Milan, Italy

Correspondence to Anthony Manyara; Anthony.Manyara@glasgow.ac.uk

For peer review only

For peer review only - http://bmjopen.bmj.com/site/about/guidelines.xhtml

Abstract

Introduction

Using a surrogate endpoint as a substitute for a primary patient-relevant outcome enables randomised controlled trials (RCTs) to be conducted more efficiently, i.e., with shorter time, smaller sample size, and lower cost. However, there is currently no consensus-driven guideline for the reporting of RCTs using a surrogate endpoint as a primary outcome; therefore, we seek to develop SPIRIT (Standard Protocol Items: Recommendations for Interventional Trials) and CONSORT (Consolidated Standards of Reporting Trials) extensions to improve the design and reporting of these trials. As an initial step, scoping and targeted reviews will identify potential items for inclusion in the extensions and participants to contribute to a Delphi consensus process.

Methods and analysis

The scoping review will search and include literature reporting on the current understanding, limitations, and guidance on using surrogate endpoints in trials. Relevant literature will be identified through: 1) bibliographic databases; 2) grey literature; 3) handsearching of reference lists; and 4) solicitation from experts. Data from eligible records will be thematically analysed into potential items for inclusion in extensions. The targeted review will search for RCT reports and protocols published from 2017-2021 in six high impact general medical journals. Trial corresponding author contacts will be listed as potential participants for the Delphi exercise.

Ethics and dissemination

Ethical approval is not required. The reviews will support the development of SPIRIT and CONSORT extensions for reporting surrogate primary endpoints (surrogate endpoint as the primary outcome) The findings will be published in open-access publications.

This review has been prospectively registered in the OSF Registration DOI: 10.17605/OSF.IO/WP3QH.

Keywords: Surrogate endpoints, randomised controlled trials, Reporting guidelines

For peer review only - http://bmjopen.bmj.com/site/about/guidelines.xhtml

Strengths and limitations of this study

- Our scoping review will use rigorous methods to identify literature using multiple sources with no restriction to regions or time periods.
- The targeted review will identify recent randomised controlled trials that have used surrogate primary endpoints from six high impact journals.
- Due to lack of resources for translation, we will only include records in English or Italian.
- Using a purposively selected set of journals for the targeted review means that our review of recent RCT protocols and trials is not exhaustive and may lack generalisability.

For peer review only

For peer review only - http://bmjopen.bmj.com/site/about/guidelines.xhtml

Introduction

Randomised controlled trials (RCTs), that are well designed, conducted, and reported, provide rigorous scientific evidence for evaluating the effectiveness and safety of interventions intended to impact health [1 2]. Nevertheless, to meet the scientific, ethical, and regulatory requirements, the conduct and delivery of RCTs is becoming increasingly resource and time-intensive [3], with median cost estimates of up to US$ 21.4 million for phase three trials [4]. The use of a surrogate endpoint as a substitute for a primary final patient relevant outcome [5] provides a potentially attractive solution for improving efficiency of RCTs, i.e., shorter follow up, smaller sample size, and, as a result, lower cost.

A key rationale for the use of a surrogate endpoint is that the intervention effect on the surrogate fully captures the intervention effect on the final patient relevant outcome [6]. Consideration of surrogate endpoints in RCTs has traditionally focused on the regulatory setting for pharmaceuticals and whether biomarkers are "likely to predict" patient-centred outcomes of interest (e.g., systolic blood pressure for stroke, low density lipoprotein cholesterol for myocardial infarction, and HIV viral load for development of AIDS). However, it is important to acknowledge a more wider application in RCTs of intermediate outcomes that are believed to capture the causal pathway through which pharmaceutical, surgical, organizational or public health interventions impact the ultimate patient-relevant outcome (e.g., hospice enrolment for mortality with an intervention aimed at improving end of life care [7]; fruit and vegetable consumption for cardiovascular events for a behavioural intervention designed to improve cardiovascular risk [8]).

Despite the potential appeal of surrogate endpoints in RCTs, their use in clinical and policy decision making remains controversial. An empirical analysis has found that RCTs using a surrogate endpoint primary outcome typically report 46% larger treatment effects compared to RCTs with final patient relevant primary outcomes [9]. This finding is supported by theoretical analyses [10]. Concerningly, some approvals based on surrogate endpoints have led to the 'real world' use of interventions that fail to demonstrate their predicted benefit(s) on the ultimate patient-centred outcome of interest and even more worryingly, that result in more harm than good [11 12]. Therefore, design and reporting of RCTs using surrogate endpoints should clearly convey the uncertainty and risks associated with their use. However, audits of RCTs to date have found this not to be the case. An analysis of 626 RCTs published in 2005 and 2006 found that 107 (17%) used a surrogate primary endpoint (surrogate endpoint as a

For peer review only - http://bmjopen.bmj.com/site/about/guidelines.xhtml

the primary outcome) and of these, only a third discussed whether the surrogate was a valid predictor of patient-relevant outcomes [13]. Furthermore, a review of 220 cardiovascular surrogate trials found that only 59 (27%) had evidence validating the benefits of interventions on a final patient-relevant outcome [14].

Reporting guidelines can guide design and improve the reporting of RCTs at both the protocol and report stages. Two established guidelines are: SPIRIT (Standard Protocol Items: Recommendations for Interventional Trials) 2013 statement: a 33-item checklist used to guide the drafting of RCT protocols [15]; and CONSORT (Consolidated Standards of Reporting Trials) 2010 Statement is a 25-item checklist used to improve reporting of conducted trials [16]. Yet, although SPIRIT and CONSORT (and related extensions) provide general guidance on outcome reporting, there remains no standard evidence-based reference for dealing with surrogacy of the primary endpoint. Improving transparency in the reporting of trials using surrogates would enable the evidence base for the surrogate to be more effectively scrutinised. Therefore, we aim to develop extensions to report trial protocols and reports that use surrogate primary endpoints: SPIRIT-SURROGATE and CONSORT-SURROGATE, respectively. The extensions focus on trials using surrogate endpoints as primary outcomes (including as part of a composite outcome) as these would inform trial conclusions and interpretations of results and possible approval of interventions. Our working definition of a surrogate endpoint is: 'a biomarker or intermediate outcome used to substitute and predict for a final patient relevant outcome (i.e., characteristic or variable that captures how a patient feels, functions, or how long they survive, such as the outcomes of mortality or health-related quality of life)' [5 6 17]. Additionally, reference of surrogate endpoints refers to statistically validated surrogate endpoints (e.g., change in systolic blood pressure for cardiovascular mortality in anti-hypertensive treatments) and non-validated surrogates for which there is still not convincing evidence that they are 'reasonably likely to predict health benefit' (e.g., reduction in amyloid load in Alzheimer's disease) [18 19] .To develop these extensions, we will closely follow the EQUATOR (Enhancing the QUAlity and Transparency Of health Research) network's recommended steps for developing a health research reporting guideline [20]. We have structured our project into four phases: Phase 1 (Literature reviews), Phase 2 (Delphi study), Phase 3 (Consensus meeting), and Phase 4 (Knowledge translation). This protocol outlines the activities and procedures of Phase 1 consisting of scoping and targeted reviews. The scoping review will be used to: synthesise current evidence and guidance on using surrogate endpoints to generate candidate items for potential inclusion in extensions;

For peer review only - http://bmjopen.bmj.com/site/about/guidelines.xhtml

and identify surrogate content experts for recruitment in the Delphi study (Phase 2). The primary aim of the targeted review is to identify trial investigators who have led an RCT using a surrogate endpoint to be invited to participate in the Delphi exercise. A secondary aim will be to archive identified protocols and trials and use them as a 'baseline' for future evaluation of the impact of developed extensions on the reporting practice of future RCT protocols and reports.

Methods and analysis

Scoping review

The scoping review was considered to be the most suitable knowledge synthesis approach for addressing the broad aim of this study [21]. The scoping review will be conducted using a methodological framework proposed by Arksey and O'Malley [22], and enhancements proposed to this framework by Levac et al [23] and Peters et al [24]. This will involve six stages: formulating a research question; identifying relevant studies; inclusion of studies; charting data; summarising and reporting results; and consultation [22].

Framework stage one: Formulating the research question

This scoping review seeks to identify a list of items that should be considered when reporting RCT protocols and reports which use surrogate endpoints. Therefore, our overarching research question combines a broad scope and a specific area of inquiry [23] (i.e., surrogate endpoint use): what is the current understanding, advice, and guidance on using surrogate endpoints in RCTs? Specific research questions are:

1. How are surrogate endpoints defined?
2. What are the limitations of using surrogate endpoints in RCTs?
3. When is the use of surrogate endpoints acceptable?
4. What published advice and guidance exists on reporting RCTs protocols and reports using surrogate endpoints?

There is a possibility of modification of these research questions during the literature reviewing and this will be reported when publishing the findings.

Framework stage two: Identifying relevant literature

We will adopt a search approach that balances comprehensiveness, breadth, and feasibility [23]. Relevant literature will be identified through: 1) electronic bibliographic databases (Excerpta Medica Database (EMBASE), Medical Literature Analysis and Retrieval System Online

For peer review only - http://bmjopen.bmj.com/site/about/guidelines.xhtml

(MEDLINE), Cochrane Methodology Register); 2) Grey literature (Google and relevant website search); 3) handsearching of reference lists; and 4) solicitation for additional literature from expert colleagues [22-24].

Electronic databases and grey literature search will be supported by an experienced information specialist (VW). We have developed an initial search strategy for MEDLINE and EMBASE which combines "surrogate endpoints", "guidelines", and "trials" related search terms (see Table 1 and 2 in Supplementary File 1). This strategy was checked for validity against four highly cited articles (>50 citations) that answer either of our specific research questions [9 13 25 26].

For grey literature, search strategies will be modified for each of the websites and for each strategy, the search terms and the number of results retrieved and/or screened will be recorded [27]. Table 3 in Supplementary File 1 shows search strategies to be used in the Google search engine and in some of the relevant websites. Generally, the strategies will include combination of search terms (e.g., "surrogate endpoints" AND "guidance") in Google advanced search; broad searches (e.g., surrogate endpoints) using the website search function; and browsing for websites without a search function. For large websites (e.g., www.ema.europe.eu), Google advanced search will be used, and search limited to the website URL. The first 100 hits in each search will be screened for eligibility to balance between feasibility and relevancy of records [27]. One reviewer will screen searches on the Google search engine or websites using title and, if present, any short text underneath.

All reference lists of included full texts will be screened to identify relevant records. We will solicit for additional resources from surrogate and outcome measurement experts including authors of a recent scoping review (on "outcome reporting recommendations for trial protocols and reports") which identified eight documents that focused on reporting recommendations for surrogate outcomes [28].

Framework stage three: Literature selection

Databases search results will be exported to Endnote version X9 for the removal of duplicates. The remaining records will be exported to Covidence [29] for eligibility screening based on title, abstract, and full-text reading by two reviewers. Title and abstract screening of grey literature will be done in respective websites by one reviewer and full-text screening done from HTML files by two reviewers.

For peer review only - http://bmjopen.bmj.com/site/about/guidelines.xhtml

Once full-text screening has been concluded, reviewers will hand search reference lists of all included full-texts for relevant records. The identified records combined with those supplied from experts will undergo full-text screening. Records will be eligible for inclusion if they report findings relevant to any research question. While we will mainly include records that are peer-reviewed literature, academic or regulatory grey literature (e.g., white papers), reviewers will make judgements on inclusion of other records (e.g., conference abstracts) based on relevance to review questions and trustworthiness of evidence presented. We will not restrict our inclusion of literature to regions or time periods. However, we will only include records in English or Italian due to lack of resources for translation. Disagreements between reviewers will be resolved by consensus or, if necessary, involving a third reviewer.

Framework stage four: Charting the findings

The following data will be extracted: author (and contact of corresponding author), publication year, country, author affiliation category (e.g., academic, regulatory body, patient/public forums), record type (e.g., review article, commentary, regulatory guidance), research area if specified, funding if stated, and findings relevant to research questions (i.e., definition, limitations, acceptability, guidance on surrogate endpoints use). A pilot will be undertaken to check if the data extraction template needs modification. All data extraction will be done by one reviewer. At the start of extraction, a subset of extracted data (~10% of records) will be checked for accuracy by a second reviewer and if accurate the first reviewer will proceed to extract in all other records.

Framework stage five: Synthesis and reporting the findings

All analysis will be done in Microsoft Excel. Descriptive data (i.e., publication year, country/region, author affiliation category, record type) will be analysed using counts and percentages and presented in tables, graphs or as text. Data related to research questions (e.g., key messages/advice/guidelines on surrogate endpoints use) will be collated verbatim under each research question. A simple form of thematic analysis [30] will then be used to synthesise data. Two reviewers will independently read the collated data under each research question and for each record, summarise it into: 1) item(s) to be considered when reporting protocols and trials using surrogate endpoints; and 2) whether the items are new or modifications to the SPIRIT and/or CONSORT checklist items and for new items, the section of the checklist where they should be reported. The reviewers will then meet for a virtual workshop to discuss and agree on items and their designated sections of the checklist. We will report the findings

For peer review only - http://bmjopen.bmj.com/site/about/guidelines.xhtml

in an open-access peer reviewed publication using the Preferred Reporting Items for Systematic reviews and Meta-Analysis for Scoping Reviews (PRISMA-ScR) [31].

Framework stage six: Consultation exercise

The aim of consultation is to share scoping review findings with stakeholders so as to identify additional relevant resources and valuable insights that the scoping review findings may have missed [22]. Nevertheless, it is important to specify when, how, and why to do consultation, the types of stakeholder involved, and how to integrate the information with review findings [23]. We will use preliminary review findings to seek insights, through virtual meetings, from Public and Patient Involvement (PPI) representatives on the identified items for reporting surrogate endpoints. Our project PPI Lead (DS) will coordinate consultation with PPI representatives, and this will offer an opportunity for knowledge transfer and exchange. Additionally, we will invite our multidisciplinary expert advisory Executive Committee members (see Acknowledgement) and the MRC-NIHR Trials Methodology Research Partnership Outcomes Working Group (www.methodologyhubs.mrc.ac.uk/), specifically the Surrogate Outcomes subgroup, to comment on any additional resources, items, and perspectives not included in the preliminary findings. Review comments on the preliminary findings document or detailed notes taken during consultation meetings will be used in summarising and integrating suggested items into the review findings.

Targeted review

The targeted reviews are intended to identify trial investigators who have led an RCT assessing a surrogate endpoint and protocols and trials that have a primary surrogate endpoint [9]. MEDLINE through PubMed will be searched for RCTs published in the last five years (2017-2021) in six high impact general medical journals: *Annals of Internal Medicine*, *BMJ*, *Journal of the American Medical Association*, *New England Journal of Medicine*, *Lancet*, and *PLoS Medicine.* Use of general medical journals allows for inclusion of records across a range of clinical areas. Given the focus of the project on reporting guidelines for trial protocols, we also will search two journals widely used for publishing RCT protocols: *BMJ Open* and *Trials*. We will include trial protocols and reports that use outcomes that meet our working definition of surrogate endpoints.

All identified protocols and trials will be exported to Endnote version X9 for the removal of duplicates and exported to Covidence [29] for eligibility screening. Given the primary objective of this review is to identify trial investigators who have used surrogate endpoints, screening

For peer review only - http://bmjopen.bmj.com/site/about/guidelines.xhtml

will be limited to titles and abstracts. Two reviewers will screen all records and include those protocols and randomised trials that use surrogate primary endpoints and report intervention studies. A more in-depth screening and analysis of the full texts will be done as part of an upcoming project, acting as a baseline to evaluate the impact of the extensions (post-publication) on the reporting of RCT protocols and trials.

From the included records, one reviewer will extract the title, journal, year of publication, research area, corresponding author name, institutional affiliation, and email address. These data will be used to sample and recruit participants for the Delphi study (Phase 2 of the project).

Patient and public involvement

One of the project team members (DS) is a leading PPI advocate who has been involved in health research at local, national, and international level. As outlined, PPI will be integrated in stage six of the scoping review. We are additionally exploring how patients and the public can be meaningfully involved in this project.

Limitations

Although we will use four strategies in our scoping review searches, our inclusion will be limited to records in English and Italian language hence exclusion of non-English/Italian literature. Nevertheless, our review does not aim to be exhaustive but to identify important items for consideration when using surrogate endpoints and it is highly likely items synthesised from records in the English and Italian language would be transferable to other settings. Using an approach of a purposively selected set of journals means our targeted review of recent RCT protocols and trials is not exhaustive and may lack generalisability.

Ethics and dissemination

The reviews do not require ethics approval. The reviews findings will be disseminated through conference presentations and open-access publications.

Contributorship Statement

PD, CJW, AY, RT, and OC were involved in funding acquisition. AMM, PD, DS, CJW, AY, RT, and OC were involved in the initial phases of study conception and design. AMM, VW, RT, and OC were involved in design of the search strategy and responsible for the first draft

For peer review only - http://bmjopen.bmj.com/site/about/guidelines.xhtml

of the manuscript. PD, DS, CJW, and AY reviewed the first draft, and all authors approved the final version.

Competing Interests

None to declare.

Funding

The development of SPIRIT and CONSORT extensions has been funded by the Medical Research Council (grant number MR/V038400/1).

Acknowledgments

We acknowledge our advisory Executive Committee members who will oversight the project and contribute to the consultation stage of the scoping review: Professor Joseph Ross (Chair of the Executive Committee); Professor Martin Offringa; Dr. Nancy Butcher; Professor An-Wen Chan; Professor Gary Collins; Professor Sylwia Bujkiewicz; Dr Dalia Dawoud; and Dr Mario Ouwens.

References

1. Akobeng AK. Understanding randomised controlled trials. *Archives of Disease in Childhood* 2005;90(8):840-44. doi: 10.1136/adc.2004.058222
2. Hariton E, Locascio JJ. Randomised controlled trials - the gold standard for effectiveness research: Study design: randomised controlled trials. *BJOG* 2018;125(13):1716-16. doi: 10.1111/1471-0528.15199 [published Online First: 2018/06/19]
3. Umscheid CA, Margolis DJ, Grossman CE. Key concepts of clinical trials: a narrative review. *Postgraduate medicine* 2011;123(5):194-204. doi: 10.3810/pgm.2011.09.2475
4. Martin L, Hutchens M, Hawkins C, et al. How much do clinical trials cost? *Nature Reviews Drug Discovery* 2017;16(6):381-82. doi: 10.1038/nrd.2017.70
5. Biomarkers Definitions Working Group. Biomarkers and surrogate endpoints: Preferred definitions and conceptual framework. *Clinical Pharmacology & Therapeutics* 2001;69(3):89-95. doi: https://doi.org/10.1067/mcp.2001.113989
6. DeMets DL, Psaty BM, Fleming TR. When Can Intermediate Outcomes Be Used as Surrogate Outcomes? *Jama* 2020;323(12):1184-85. doi: 10.1001/jama.2020.1176 [published Online First: 2020/02/28]
7. Casarett D, Karlawish J, Morales K, et al. Improving the use of hospice services in nursing homes: a randomized controlled trial. *Jama* 2005;294(2):211-7. doi: 10.1001/jama.294.2.211 [published Online First: 2005/07/15]
8. Domke A, Keller J, Heuse S, et al. Immediate effects of a very brief planning intervention on fruit and vegetable consumption: A randomized controlled trial. *Applied Psychology: Health and Well-Being* 2021;13(2):377-93. doi: https://doi.org/10.1111/aphw.12254
9. Ciani O, Buyse M, Garside R, et al. Comparison of treatment effect sizes associated with surrogate and final patient relevant outcomes in randomised controlled trials: meta-epidemiological study. *BMJ : British Medical Journal* 2013;346:f457. doi: 10.1136/bmj.f457

For peer review only - http://bmjopen.bmj.com/site/about/guidelines.xhtml

10. Walter SD, Sun X, Heels-Ansdell D, et al. Treatment effects on patient-important outcomes can be small, even with large effects on surrogate markers. *J Clin Epidemiol* 2012;65(9):940-5. doi: 10.1016/j.jclinepi.2012.02.012 [published Online First: 2012/05/23]
11. Grimes DA, Schulz KF. Surrogate end points in clinical research: hazardous to your health. *Obstet Gynecol* 2005;105(5 Pt 1):1114-8. doi: 10.1097/01.aog.0000157445.67309.19 [published Online First: 2005/05/03]
12. Thomas R. Fleming, David L. DeMets. Surrogate End Points in Clinical Trials: Are We Being Misled? *Annals of Internal Medicine* 1996;125(7):605-13. doi: 10.7326/0003-4819-125-7-199610010-00011 %m 8815760
13. la Cour JL, Brok J, Gøtzsche PC. Inconsistent reporting of surrogate outcomes in randomised clinical trials: cohort study. *BMJ* 2010;341:c3653-c53. doi: 10.1136/bmj.c3653
14. Bikdeli B, Punnanithinont N, Akram Y, et al. Two Decades of Cardiovascular Trials With Primary Surrogate Endpoints: 1990-2011. *J Am Heart Assoc* 2017;6(3):e005285. doi: 10.1161/JAHA.116.005285
15. Chan AW, Tetzlaff JM, Altman DG, et al. SPIRIT 2013 statement: defining standard protocol items for clinical trials. *Ann Intern Med* 2013;158(3):200-7. doi: 10.7326/0003-4819-158-3-201302050-00583 [published Online First: 2013/01/09]
16. Schulz KF, Altman DG, Moher D, et al. CONSORT 2010 Statement: updated guidelines for reporting parallel group randomised trials. *BMC Medicine* 2010;8(1):18. doi: 10.1186/1741-7015-8-18
17. Ciani O, Grigore B, Blommestein H, et al. Validity of Surrogate Endpoints and Their Impact on Coverage Recommendations: A Retrospective Analysis across International Health Technology Assessment Agencies. *Med Decis Making* 2021;41(4):439-52. doi: 10.1177/0272989x21994553 [published Online First: 2021/03/16]
18. FDA-NIH Biomarker Working Group. BEST (Biomarkers, endpoints, and other tools) resource [Internet]. 2021
19. FDA. Table of Surrogate Endpoints That Were the Basis of Drug Approval or Licensure 2022 [Available from: https://www.fda.gov/drugs/development-resources/table-surrogate-endpoints-were-basis-drug-approval-or-licensure accessed 20/07/2022.
20. Moher D, Schulz KF, Simera I, et al. Guidance for developers of health research reporting guidelines. *PLoS medicine* 2010;7(2):e1000217. doi: 10.1371/journal.pmed.1000217 [published Online First: 2010/02/20]
21. Munn Z, Peters MDJ, Stern C, et al. Systematic review or scoping review? Guidance for authors when choosing between a systematic or scoping review approach. *BMC Medical Research Methodology* 2018;18(1):143. doi: 10.1186/s12874-018-0611-x
22. Arksey H, O'Malley L. Scoping studies: towards a methodological framework. *International Journal of Social Research Methodology* 2005;8(1):19-32. doi: 10.1080/1364557032000119616
23. Levac D, Colquhoun H, O'Brien KK. Scoping studies: advancing the methodology. *Implementation Science* 2010;5(1):69. doi: 10.1186/1748-5908-5-69
24. Peters MDJ, Godfrey CM, Khalil H, et al. Guidance for conducting systematic scoping reviews. *JBI Evidence Implementation* 2015;13(3)
25. Fleming TR, DeMets DL. Surrogate end points in clinical trials: are we being misled? *Ann Intern Med* 1996;125(7):605-13. doi: 10.7326/0003-4819-125-7-199610010-00011 [published Online First: 1996/10/01]
26. Kemp R, Prasad V. Surrogate endpoints in oncology: when are they acceptable for regulatory and clinical decisions, and are they currently overused? *BMC medicine* 2017;15(1):1-7.
27. Godin K, Stapleton J, Kirkpatrick SI, et al. Applying systematic review search methods to the grey literature: a case study examining guidelines for school-based breakfast programs in Canada. *Systematic reviews* 2015;4:138-38. doi: 10.1186/s13643-015-0125-0
28. Butcher NJ, Mew EJ, Monsour A, et al. Outcome reporting recommendations for clinical trial protocols and reports: a scoping review. *Trials* 2020;21(1):620. doi: 10.1186/s13063-020-04440-w
29. Covidence. Covidence systematic review software, Veritas Health Innovation, Melbourne, Australia.

For peer review only - http://bmjopen.bmj.com/site/about/guidelines.xhtml

30. Braun V, Clarke V. Using thematic analysis in psychology. *Qualitative research in psychology* 2006;3(2):77-101.
31. McGowan J, Straus S, Moher D, et al. Reporting scoping reviews-PRISMA ScR extension. *J Clin Epidemiol* 2020;123:177-79. doi: 10.1016/j.jclinepi.2020.03.016 [published Online First: 2020/04/02]

For peer review only

For peer review only - http://bmjopen.bmj.com/site/about/guidelines.xhtml

Supplementary File 1

Supplementary Table 1: Search strategy for MEDLINE

1	*Endpoint Determination/mt, st [Methods, Standards]
2	(Surrogate adj2 (measure* or outcome* or endpoint* or end point*)).ab,kw,ti.
3	(Surrogate and (controlled or trials)).ti.
4	(Endpoint adj1 determination).ab,kw,ti.
5	(Intermediate adj2 (outcome* or endpoint* or end point*)).ab,kw,ti.
6	1 or 2 or 3 or 4 or 5
7	Guideline Adherence/
8	Practice Guidelines as Topic/
9	Guidelines as Topic/
10	Checklist/
11	(Comparison or Regulation or regulatory or Policy or Decisions or Recommendation or Decision making or limitation* or understanding or reporting or critique or concept or conceptual or Validation or validity or recommendation or recommendations or guidance or advice or guideline* or guide line* or checklist or checklists or check list* or standard or standards or requirement* or instruction*).ti.
12	7 or 8 or 9 or 10 or 11
13	6 and 12
14	Clinical Trials as Topic/
15	Cohort Studies/
16	Randomized Controlled Trials as Topic/
17	"Reproducibility of Results"/
18	Research Design/
19	Data Collection/
20	Drug Approval/
21	Treatment Outcome/
22	Outcome Assessment, Health Care/
23	(outcomes or regulation).ti.
24	(clinical adj1 outcome assessment).ti.
25	clinical trials.ti.
26	14 or 15 or 16 or 17 or 18 or 19 or 20 or 21 or 22 or 23 or 24
27	13 and 26

For peer review only - http://bmjopen.bmj.com/site/about/guidelines.xhtml

Supplementary Table 2: Search strategy for EMBASE

1	(Surrogate adj2 (measure* or outcome* or endpoint* or end point*)).ab,kw,ti.
2	(Surrogate and (controlled or trials)).ti.
3	(Endpoint adj1 determination).ab,kw,ti.
4	(Intermediate adj2 (outcome* or endpoint* or end point*)).ab,kw,ti.
5	1 or 2 or 3 or 4
6	protocol compliance/
7	practice guideline/
8	checklist/
9	(Comparison or Regulation or regulatory or Policy or Decisions or Recommendation or Decision making or limitation* or understanding or reporting or critique or concept or conceptual or Validation or validity or recommendation or recommendations or guidance or advice or guideline* or guide line* or checklist or checklists or check list* or standard or standards or requirement* or instruction*).ti.
10	6 or 7 or 8 or 9
11	5 and 10
12	"clinical trial (topic)"/
13	cohort analysis/
14	"randomized controlled trial (topic)"/
15	reproducibility/
16	methodology/
17	information processing/
18	drug approval/
19	treatment outcome/
20	outcome assessment/
21	(outcomes or regulation).ti.
22	(clinical adj1 outcome assessment).ti.
23	clinical trials.ti.
24	12 or 13 or 14 or 15 or 16 or 17 or 18 or 19 or 20 or 21 or 22 or 23
25	11 and 24

For peer review only

For peer review only - http://bmjopen.bmj.com/site/about/guidelines.xhtml

Supplementary Table 3: Search strategy for Grey literature

Search strategy for grey literature. For each unique search, 100 hits will be reviewed for eligibility.

Source	Search strategy
**Google search engine**	Advanced search option (www.google.co.uk/advanced_search) will be used. Searches will combine terms appearing in the titles of for example:<br>• "surrogate endpoints" AND "recommendation" OR "guidance" OR "considerations"<br>• "clinical endpoints" AND "recommendation" OR "guidance" OR "considerations"
**Examples of relevant websites**	
**FDA (www.fda.gov)**	Use a search function to do a broad search using terms such as "surrogate endpoints", "clinical endpoints"
**MHRA (www.gov.uk/government/organisations/medicines-and-healthcare-products-regulatory-agency)**	No search option website will be browsed.
**European Medicines Agency (www.ema.europe.eu)**	Given its a large website, Advance Google search will be used, and search limited by the search by URL
**COMET initiative (www.comet-initiative.org)**	Searches done by options provided by the website. For example: by Method= Literature review; by Study Type =Commentary, COS methods research

For peer review only - http://bmjopen.bmj.com/site/about/guidelines.xhtml

Protocol for scoping and targeted reviews to support development of SPIRIT and CONSORT extensions for randomised controlled trials with surrogate primary endpoints

Journal:	*BMJ Open*
Manuscript ID	bmjopen-2022-062798.R2
Article Type:	Protocol
Date Submitted by the Author:	15-Sep-2022
Complete List of Authors:	Manyara, Anthony; University of Glasgow, MRC/CSO Social and Public Health Sciences Unit, Institute of Health and Wellbeing<br>Davies, Philippa ; University of Bristol, Population Health Sciences, Bristol Medical School<br>Stewart, Derek; Patient and Public Involvement lead<br>Wells, Valerie; University of Glasgow, MRC/CSO Social and Public Health Sciences Unit, Institute of Health and Wellbeing<br>Weir, Christopher; University of Edinburgh, Edinburgh Clinical Trials Unit, Usher Institute<br>Young, Amber; University Hospitals Bristol NHS Foundation Trust, Paediatric Anaesthesia; University of Bristol, Population Health Sciences, Bristol Medical School<br>Taylor, Rod; University of Glasgow, MRC/CSO Social and Public Health Sciences Unit, Institute of Health and Wellbeing; University of Glasgow, Robertson Centre for Biostatistics, Institute of Health and Well Being<br>Ciani, Oriana ; SDA Bocconi School of Management
**Primary Subject Heading**:	Research methods
Secondary Subject Heading:	Epidemiology, Patient-centred medicine, Medical publishing and peer review
Keywords:	Protocols & guidelines < HEALTH SERVICES ADMINISTRATION & MANAGEMENT, EPIDEMIOLOGY, PUBLIC HEALTH

Protocol for scoping and targeted reviews to support development of SPIRIT and CONSORT extensions for randomised controlled trials with surrogate primary endpoints

Anthony Muchai Manyara[1], Philippa Davies[2], Derek Stewart[3], Valerie Wells[1], Christopher J Weir[4], Amber Young[2], Rod S Taylor[1,4], Oriana Ciani[5]

1 MRC/CSO Social and Public Health Sciences Unit, Institute of Health and Wellbeing, University of Glasgow, UK

2 Population Health Sciences, Bristol Medical School, University of Bristol, UK

3 Patient and Public Involvement lead, UK

4 Edinburgh Clinical Trials Unit, Usher Institute, University of Edinburgh, UK

5 Robertson Centre for Biostatistics, Institute of Health and Well Being, University of Glasgow, UK

6 SDA Bocconi School of Management, Milan, Italy

Correspondence to Anthony Manyara; Anthony.Manyara@glasgow.ac.uk

For peer review only

For peer review only - http://bmjopen.bmj.com/site/about/guidelines.xhtml

Abstract

Introduction

Using a surrogate endpoint as a substitute for a primary patient-relevant outcome enables randomised controlled trials (RCTs) to be conducted more efficiently, i.e., with shorter time, smaller sample size, and lower cost. However, there is currently no consensus-driven guideline for the reporting of RCTs using a surrogate endpoint as a primary outcome; therefore, we seek to develop SPIRIT (Standard Protocol Items: Recommendations for Interventional Trials) and CONSORT (Consolidated Standards of Reporting Trials) extensions to improve the design and reporting of these trials. As an initial step, scoping and targeted reviews will identify potential items for inclusion in the extensions and participants to contribute to a Delphi consensus process.

Methods and analysis

The scoping review will search and include literature reporting on the current understanding, limitations, and guidance on using surrogate endpoints in trials. Relevant literature will be identified through: 1) bibliographic databases; 2) grey literature; 3) handsearching of reference lists; and 4) solicitation from experts. Data from eligible records will be thematically analysed into potential items for inclusion in extensions. The targeted review will search for RCT reports and protocols published from 2017-2021 in six high impact general medical journals. Trial corresponding author contacts will be listed as potential participants for the Delphi exercise.

Ethics and dissemination

Ethical approval is not required. The reviews will support the development of SPIRIT and CONSORT extensions for reporting surrogate primary endpoints (surrogate endpoint as the primary outcome) The findings will be published in open-access publications.

This review has been prospectively registered in the OSF Registration DOI: 10.17605/OSF.IO/WP3QH.

Keywords: Surrogate endpoints, randomised controlled trials, Reporting guidelines

For peer review only - http://bmjopen.bmj.com/site/about/guidelines.xhtml

Strengths and limitations of this study

- Our scoping review will use rigorous methods to identify literature using multiple sources with no restriction to regions or time periods.
- The targeted review will identify recent randomised controlled trials that have used surrogate primary endpoints from six high impact journals.
- Due to lack of resources for translation, we will only include records in English or Italian.
- Using a purposively selected set of journals for the targeted review means that our review of recent RCT protocols and trials is not exhaustive and may lack generalisability.

For peer review only

For peer review only - http://bmjopen.bmj.com/site/about/guidelines.xhtml

Introduction

Randomised controlled trials (RCTs), that are well designed, conducted, and reported, provide rigorous scientific evidence for evaluating the effectiveness and safety of interventions intended to impact health [1 2]. Nevertheless, to meet the scientific, ethical, and regulatory requirements, the conduct and delivery of RCTs is becoming increasingly resource and time-intensive [3], with median cost estimates of up to US$ 21.4 million for phase three trials [4]. The use of a surrogate endpoint as a substitute for a primary final patient relevant outcome [5] provides a potentially attractive solution for improving efficiency of RCTs, i.e., shorter follow up, smaller sample size, and, as a result, lower cost.

A key rationale for the use of a surrogate endpoint is that the intervention effect on the surrogate fully captures the intervention effect on the final patient relevant outcome [6]. Consideration of surrogate endpoints in RCTs has traditionally focused on the regulatory setting for pharmaceuticals and whether biomarkers are "likely to predict" patient-centred outcomes of interest (e.g., systolic blood pressure for stroke, low density lipoprotein cholesterol for myocardial infarction, and HIV viral load for development of AIDS). However, it is important to acknowledge a more wider application in RCTs of intermediate outcomes that are believed to capture the causal pathway through which pharmaceutical, surgical, organizational or public health interventions impact the ultimate patient-relevant outcome (e.g., hospice enrolment for mortality with an intervention aimed at improving end of life care [7]; fruit and vegetable consumption for cardiovascular events for a behavioural intervention designed to improve cardiovascular risk [8]). To be regarded as a valid surrogate endpoint, a biomarker or intermediate outcome is required: 1) to reliably predict the PRFO in individual trial participants ('individual level' or 'patient-level' surrogacy); and 2) the intervention effect on the surrogate endpoint should reliably predict the intervention effect on the PRFO ('trial-level' surrogacy) based on evidence from meta-analyses of RCT data on both outcomes[9 10]. Statistical surrogate validation uses various statistical methods, including meta-analyses of RCT aggregate and/or individual patient data[11 12], principal stratification [13], causal inference [14 15], bivariate network meta-analysis methods[16 17] and information theory [18]. However, surrogate validation should extend beyond statistical validity to include a multifaceted approach comprising of biological plausibility rationale and "face validity" of the surrogates in trials[19].

For peer review only - http://bmjopen.bmj.com/site/about/guidelines.xhtml

Despite the potential appeal of surrogate endpoints in RCTs, their use in clinical and policy decision making remains controversial. An empirical analysis has found that RCTs using a surrogate endpoint primary outcome typically report 46% larger treatment effects compared to RCTs with final patient relevant primary outcomes [20]. This finding is supported by theoretical analyses [21]. Concerningly, some approvals based on surrogate endpoints have led to the 'real world' use of interventions that fail to demonstrate their predicted benefit(s) on the ultimate patient-centred outcome of interest and even more worryingly, that result in more harm than good [22 23]. Therefore, design and reporting of RCTs using surrogate endpoints should clearly convey the uncertainty and risks associated with their use. However, audits of RCTs to date have found this not to be the case. An analysis of 626 RCTs published in 2005 and 2006 found that 107 (17%) used a surrogate primary endpoint (surrogate endpoint as a the primary outcome) and of these, only a third discussed whether the surrogate was a valid predictor of patient-relevant outcomes [24]. Furthermore, a review of 220 cardiovascular surrogate trials found that only 59 (27%) had evidence validating the benefits of interventions on a final patient-relevant outcome [25].

Reporting guidelines can guide design and improve the reporting of RCTs at both the protocol and report stages. Two established guidelines are: SPIRIT (Standard Protocol Items: Recommendations for Interventional Trials) 2013 statement: a 33-item checklist used to guide the drafting of RCT protocols [26]; and CONSORT (Consolidated Standards of Reporting Trials) 2010 Statement is a 25-item checklist used to improve reporting of conducted trials [27]. Yet, although SPIRIT and CONSORT (and related extensions) provide general guidance on outcome reporting, there remains no standard evidence-based reference for dealing with surrogacy of the primary endpoint. Improving transparency in the reporting of trials using surrogates would enable the evidence base for the surrogate to be more effectively scrutinised. Therefore, we aim to develop extensions to report trial protocols and reports that use surrogate primary endpoints: SPIRIT-SURROGATE and CONSORT-SURROGATE, respectively. The extensions focus on trials using surrogate endpoints as primary outcomes (including as part of a composite outcome) as these would inform trial conclusions and interpretations of results and possible approval of interventions. Our working definition of a surrogate endpoint is: 'a biomarker or intermediate outcome used to substitute and predict for a final patient relevant outcome (i.e., characteristic or variable that captures how a patient feels, functions, or how long they survive, such as the outcomes of mortality or health-related quality of life)' [5 6 28]. Additionally, reference of surrogate endpoints in this project refers to

For peer review only - http://bmjopen.bmj.com/site/about/guidelines.xhtml

statistically validated surrogate endpoints (e.g., change in systolic blood pressure for cardiovascular mortality in anti-hypertensive treatments[29 30], disease-free survival (and progression free survival in advanced disease) in colorectal cancer[31]) and non-validated surrogates for which are 'reasonably likely to predict health benefit' (e.g., reduction in amyloid load in Alzheimer's disease)[29 32] To develop these extensions, we will closely follow the EQUATOR (Enhancing the QUAlity and Transparency Of health Research) network's recommended steps for developing a health research reporting guideline [33]. We have structured our project into four phases: Phase 1 (Literature reviews), Phase 2 (Delphi study), Phase 3 (Consensus meeting), and Phase 4 (Knowledge translation). This protocol outlines the activities and procedures of Phase 1 consisting of scoping and targeted reviews. The scoping review will be used to: synthesise current evidence and guidance on using surrogate endpoints to generate candidate items for potential inclusion in extensions; and identify surrogate content experts for recruitment in the Delphi study (Phase 2). The primary aim of the targeted review is to identify trial investigators who have led an RCT using a surrogate endpoint to be invited to participate in the Delphi exercise. A secondary aim will be to archive identified protocols and trials and use them as a 'baseline' for future evaluation of the impact of developed extensions on the reporting practice of future RCT protocols and reports.

Methods and analysis

Scoping review

The scoping review was considered to be the most suitable knowledge synthesis approach for addressing the broad aim of this study [34]. The scoping review will be conducted using a methodological framework proposed by Arksey and O'Malley [35], and enhancements proposed to this framework by Levac et al [36] and Peters et al [37]. This will involve six stages: formulating a research question; identifying relevant studies; inclusion of studies; charting data; summarising and reporting results; and consultation [35].

Framework stage one: Formulating the research question

This scoping review seeks to identify a list of items that should be considered when reporting RCT protocols and reports which use surrogate endpoints. Therefore, our overarching research question combines a broad scope and a specific area of inquiry [36] (i.e., surrogate endpoint use): what is the current understanding, advice, and guidance on using surrogate endpoints in RCTs? Specific research questions are:

1. How are surrogate endpoints defined?

For peer review only - http://bmjopen.bmj.com/site/about/guidelines.xhtml

2. What are the limitations of using surrogate endpoints in RCTs?
3. When is the use of surrogate endpoints acceptable?
4. What published advice and guidance exists on reporting RCTs protocols and reports using surrogate endpoints?

There is a possibility of modification of these research questions during the literature reviewing and this will be reported when publishing the findings.

Framework stage two: Identifying relevant literature

We will adopt a search approach that balances comprehensiveness, breadth, and feasibility [36]. Relevant literature will be identified through: 1) electronic bibliographic databases (Excerpta Medica Database (EMBASE), Medical Literature Analysis and Retrieval System Online (MEDLINE), Cochrane Methodology Register); 2) Grey literature (Google and relevant website search); 3) handsearching of reference lists; and 4) solicitation for additional literature from expert colleagues [35-37].

Electronic databases and grey literature search will be supported by an experienced information specialist (VW). We have developed an initial search strategy for MEDLINE and EMBASE which combines "surrogate endpoints", "guidelines", and "trials" related search terms (see Table 1 and 2 in Supplementary File 1). This strategy was checked for validity against four highly cited articles (>50 citations) that answer either of our specific research questions [20 24 38 39].

For grey literature, search strategies will be modified for each of the websites and for each strategy, the search terms and the number of results retrieved and/or screened will be recorded [40]. Table 3 in Supplementary File 1 shows search strategies to be used in the Google search engine and in some of the relevant websites. Generally, the strategies will include combination of search terms (e.g., "surrogate endpoints" AND "guidance") in Google advanced search; broad searches (e.g., surrogate endpoints) using the website search function; and browsing for websites without a search function. For large websites (e.g., www.ema.europe.eu), Google advanced search will be used, and search limited to the website URL. The first 100 hits in each search will be screened for eligibility to balance between feasibility and relevancy of records [40]. One reviewer will screen searches on the Google search engine or websites using title and, if present, any short text underneath.

All reference lists of included full texts will be screened to identify relevant records. We will solicit for additional resources from surrogate and outcome measurement experts including

For peer review only - http://bmjopen.bmj.com/site/about/guidelines.xhtml

authors of a recent scoping review (on "outcome reporting recommendations for trial protocols and reports") which identified eight documents that focused on reporting recommendations for surrogate outcomes [41].

Framework stage three: Literature selection

Databases search results will be exported to Endnote version X9 for the removal of duplicates. The remaining records will be exported to Covidence [42] for eligibility screening based on title, abstract, and full-text reading by two reviewers. Title and abstract screening of grey literature will be done in respective websites by one reviewer and full-text screening done from HTML files by two reviewers.

Once full-text screening has been concluded, reviewers will hand search reference lists of all included full-texts for relevant records. The identified records combined with those supplied from experts will undergo full-text screening. Records will be eligible for inclusion if they report findings relevant to any research question. While we will mainly include records that are peer-reviewed literature, academic or regulatory grey literature (e.g., white papers), reviewers will make judgements on inclusion of other records (e.g., conference abstracts) based on relevance to review questions and trustworthiness of evidence presented. We will not restrict our inclusion of literature to regions or time periods. However, we will only include records in English or Italian due to lack of resources for translation. Disagreements between reviewers will be resolved by consensus or, if necessary, involving a third reviewer.

Framework stage four: Charting the findings

The following data will be extracted: author (and contact of corresponding author), publication year, country, author affiliation category (e.g., academic, regulatory body, patient/public forums), record type (e.g., review article, commentary, regulatory guidance), research area if specified, funding if stated, and findings relevant to research questions (i.e., definition, limitations, acceptability, guidance on surrogate endpoints use). A pilot will be undertaken to check if the data extraction template needs modification. All data extraction will be done by one reviewer. At the start of extraction, a subset of extracted data (~10% of records) will be checked for accuracy by a second reviewer and if accurate the first reviewer will proceed to extract in all other records.

Framework stage five: Synthesis and reporting the findings

All analysis will be done in Microsoft Excel. Descriptive data (i.e., publication year, country/region, author affiliation category, record type) will be analysed using counts and

For peer review only - http://bmjopen.bmj.com/site/about/guidelines.xhtml

percentages and presented in tables, graphs or as text. Data related to research questions (e.g., key messages/advice/guidelines on surrogate endpoints use) will be collated verbatim under each research question. A simple form of thematic analysis [43] will then be used to synthesise data. Two reviewers will independently read the collated data under each research question and for each record, summarise it into: 1) item(s) to be considered when reporting protocols and trials using surrogate endpoints; and 2) whether the items are new or modifications to the SPIRIT and/or CONSORT checklist items and for new items, the section of the checklist where they should be reported. The reviewers will then meet for a virtual workshop to discuss and agree on items and their designated sections of the checklist. We will report the findings in an open-access peer reviewed publication using the Preferred Reporting Items for Systematic reviews and Meta-Analysis for Scoping Reviews (PRISMA-ScR) [44].

Framework stage six: Consultation exercise

The aim of consultation is to share scoping review findings with stakeholders so as to identify additional relevant resources and valuable insights that the scoping review findings may have missed [35]. Nevertheless, it is important to specify when, how, and why to do consultation, the types of stakeholder involved, and how to integrate the information with review findings [36]. We will use preliminary review findings to seek insights, through virtual meetings, from Public and Patient Involvement (PPI) representatives on the identified items for reporting surrogate endpoints. Our project PPI Lead (DS) will coordinate consultation with PPI representatives, and this will offer an opportunity for knowledge transfer and exchange. Additionally, we will invite our multidisciplinary expert advisory Executive Committee members (see Acknowledgement) and the MRC-NIHR Trials Methodology Research Partnership Outcomes Working Group (www.methodologyhubs.mrc.ac.uk/), specifically the Surrogate Outcomes subgroup, to comment on any additional resources, items, and perspectives not included in the preliminary findings. Review comments on the preliminary findings document or detailed notes taken during consultation meetings will be used in summarising and integrating suggested items into the review findings.

Targeted review

The targeted reviews are intended to identify trial investigators who have led an RCT assessing a surrogate endpoint and protocols and trials that have a primary surrogate endpoint [20]. MEDLINE through PubMed will be searched for RCTs published in the last five years (2017-2021) in six high impact general medical journals: *Annals of Internal Medicine*, *BMJ*, *Journal of the American Medical Association*, *New England Journal of Medicine*, *Lancet*,

For peer review only - http://bmjopen.bmj.com/site/about/guidelines.xhtml

and *PLoS Medicine.* Use of general medical journals allows for inclusion of records across a range of clinical areas. Given the focus of the project on reporting guidelines for trial protocols, we also will search two journals widely used for publishing RCT protocols: *BMJ Open* and *Trials*. We will include trial protocols and reports that use outcomes that meet our working definition of surrogate endpoints.

All identified protocols and trials will be exported to Endnote version X9 for the removal of duplicates and exported to Covidence [42] for eligibility screening. Given the primary objective of this review is to identify trial investigators who have used surrogate endpoints, screening will be limited to titles and abstracts. Two reviewers will screen all records and include those protocols and randomised trials that use surrogate primary endpoints and report intervention studies. A more in-depth screening and analysis of the full texts will be done as part of an upcoming project, acting as a baseline to evaluate the impact of the extensions (post-publication) on the reporting of RCT protocols and trials.

From the included records, one reviewer will extract the title, journal, year of publication, research area, corresponding author name, institutional affiliation, and email address. These data will be used to sample and recruit participants for the Delphi study (Phase 2 of the project).

Patient and public involvement

One of the project team members (DS) is a leading PPI advocate who has been involved in health research at local, national, and international level. As outlined, PPI will be integrated in stage six of the scoping review. We are additionally exploring how patients and the public can be meaningfully involved in this project.

Limitations

Although we will use four strategies in our scoping review searches, our inclusion will be limited to records in English and Italian language hence exclusion of non-English/Italian literature. Nevertheless, our review does not aim to be exhaustive but to identify important items for consideration when using surrogate endpoints and it is highly likely items synthesised from records in the English and Italian language would be transferable to other settings. Using an approach of a purposively selected set of journals means our targeted review of recent RCT protocols and trials is not exhaustive and may lack generalisability.

For peer review only - http://bmjopen.bmj.com/site/about/guidelines.xhtml

Ethics and dissemination

The reviews do not require ethics approval. The reviews findings will be disseminated through conference presentations and open-access publications.

Contributorship Statement

PD, CJW, AY, RT, and OC were involved in funding acquisition. AMM, PD, DS, CJW, AY, RT, and OC were involved in the initial phases of study conception and design. AMM, VW, RT, and OC were involved in design of the search strategy and responsible for the first draft of the manuscript. PD, DS, CJW, and AY reviewed the first draft and all authors approved the final version.

Competing Interests

None to declare.

Funding

The development of SPIRIT and CONSORT extensions has been funded by the Medical Research Council (PD, CJW, AY, RT, OC, grant number MR/V038400/1).

Acknowledgments

We acknowledge our advisory Executive Committee members who will oversight the project and contribute to the consultation stage of the scoping review: Professor Joseph Ross (Chair of the Executive Committee); Professor Martin Offringa; Dr. Nancy Butcher; Professor An-Wen Chan; Professor Gary Collins; Professor Sylwia Bujkiewicz; Dr Dalia Dawoud; and Dr Mario Ouwens.

References

1. Akobeng AK. Understanding randomised controlled trials. *Archives of Disease in Childhood* 2005;90(8):840-44. doi: 10.1136/adc.2004.058222
2. Hariton E, Locascio JJ. Randomised controlled trials - the gold standard for effectiveness research: Study design: randomised controlled trials. *BJOG* 2018;125(13):1716-16. doi: 10.1111/1471-0528.15199 [published Online First: 2018/06/19]

For peer review only - http://bmjopen.bmj.com/site/about/guidelines.xhtml

3. Umscheid CA, Margolis DJ, Grossman CE. Key concepts of clinical trials: a narrative review. *Postgraduate medicine* 2011;123(5):194-204. doi: 10.3810/pgm.2011.09.2475
4. Martin L, Hutchens M, Hawkins C, et al. How much do clinical trials cost? *Nature Reviews Drug Discovery* 2017;16(6):381-82. doi: 10.1038/nrd.2017.70
5. Biomarkers Definitions Working Group. Biomarkers and surrogate endpoints: Preferred definitions and conceptual framework. *Clinical Pharmacology & Therapeutics* 2001;69(3):89-95. doi: https://doi.org/10.1067/mcp.2001.113989
6. DeMets DL, Psaty BM, Fleming TR. When Can Intermediate Outcomes Be Used as Surrogate Outcomes? *Jama* 2020;323(12):1184-85. doi: 10.1001/jama.2020.1176 [published Online First: 2020/02/28]
7. Casarett D, Karlawish J, Morales K, et al. Improving the use of hospice services in nursing homes: a randomized controlled trial. *Jama* 2005;294(2):211-7. doi: 10.1001/jama.294.2.211 [published Online First: 2005/07/15]
8. Domke A, Keller J, Heuse S, et al. Immediate effects of a very brief planning intervention on fruit and vegetable consumption: A randomized controlled trial. *Applied Psychology: Health and Well-Being* 2021;13(2):377-93. doi: https://doi.org/10.1111/aphw.12254
9. Buyse M, Molenberghs G, Burzykowski T, et al. The validation of surrogate endpoints in meta-analyses of randomized experiments. *Biostatistics* 2000;1(1):49-67. doi: 10.1093/biostatistics/1.1.49 [published Online First: 2003/08/23]
10. Buyse M, Saad ED, Burzykowski T, et al. Surrogacy Beyond Prognosis: The Importance of "Trial-Level" Surrogacy. *The Oncologist* 2022;27(4):266-71. doi: 10.1093/oncolo/oyac006
11. Xie W, Halabi S, Tierney JF, et al. A Systematic Review and Recommendation for Reporting of Surrogate Endpoint Evaluation Using Meta-analyses. *JNCI Cancer Spectr* 2019;3(1):pkz002. doi: 10.1093/jncics/pkz002 [published Online First: 2019/07/31]
12. Daniels MJ, Hughes MD. Meta-analysis for the evaluation of potential surrogate markers. *Stat Med* 1997;16(17):1965-82. doi: 10.1002/(sici)1097-0258(19970915)16:17<1965::aid-sim630>3.0.co;2-m [published Online First: 1997/09/26]
13. Weir CJ, Walley RJ. Statistical evaluation of biomarkers as surrogate endpoints: a literature review. *Stat Med* 2006;25(2):183-203. doi: 10.1002/sim.2319
14. Alonso A, Van der Elst W, Molenberghs G, et al. On the relationship between the causal-inference and meta-analytic paradigms for the validation of surrogate endpoints. *Biometrics* 2015;71(1):15-24. doi: 10.1111/biom.12245 [published Online First: 2014/10/03]
15. Vanderweele TJ. Surrogate measures and consistent surrogates. *Biometrics* 2013;69(3):561-9. doi: 10.1111/biom.12071 [published Online First: 2013/10/01]
16. Bujkiewicz S, Jackson D, Thompson JR, et al. Bivariate network meta-analysis for surrogate endpoint evaluation. *Stat Med* 2019;38(18):3322-41. doi: 10.1002/sim.8187 [published Online First: 2019/05/28]
17. Papanikos T, Thompson JR, Abrams KR, et al. Bayesian hierarchical meta-analytic methods for modeling surrogate relationships that vary across treatment classes using aggregate data. *Stat Med* 2020;39(8):1103-24. doi: 10.1002/sim.8465 [published Online First: 2020/01/29]
18. Alonso A, Van der Elst W, Molenberghs G, et al. An information-theoretic approach for the evaluation of surrogate endpoints based on causal inference. *Biometrics* 2016;72(3):669-77. doi: 10.1111/biom.12483 [published Online First: 2016/02/13]
19. Green E, Yothers G, Sargent DJ. Surrogate endpoint validation: statistical elegance versus clinical relevance. *Statistical Methods in Medical Research* 2008;17(5):477-86. doi: 10.1177/0962280207081863
20. Ciani O, Buyse M, Garside R, et al. Comparison of treatment effect sizes associated with surrogate and final patient relevant outcomes in randomised controlled trials: meta-epidemiological study. *BMJ : British Medical Journal* 2013;346:f457. doi: 10.1136/bmj.f457

For peer review only

For peer review only - http://bmjopen.bmj.com/site/about/guidelines.xhtml

21. Walter SD, Sun X, Heels-Ansdell D, et al. Treatment effects on patient-important outcomes can be small, even with large effects on surrogate markers. *J Clin Epidemiol* 2012;65(9):940-5. doi: 10.1016/j.jclinepi.2012.02.012 [published Online First: 2012/05/23]
22. Grimes DA, Schulz KF. Surrogate end points in clinical research: hazardous to your health. *Obstet Gynecol* 2005;105(5 Pt 1):1114-8. doi: 10.1097/01.aog.0000157445.67309.19 [published Online First: 2005/05/03]
23. Thomas R. Fleming, David L. DeMets. Surrogate End Points in Clinical Trials: Are We Being Misled? *Annals of Internal Medicine* 1996;125(7):605-13. doi: 10.7326/0003-4819-125-7-199610010-00011 %m 8815760
24. la Cour JL, Brok J, Gøtzsche PC. Inconsistent reporting of surrogate outcomes in randomised clinical trials: cohort study. *BMJ* 2010;341:c3653-c53. doi: 10.1136/bmj.c3653
25. Bikdeli B, Punnanithinont N, Akram Y, et al. Two Decades of Cardiovascular Trials With Primary Surrogate Endpoints: 1990-2011. *J Am Heart Assoc* 2017;6(3):e005285. doi: 10.1161/JAHA.116.005285
26. Chan AW, Tetzlaff JM, Altman DG, et al. SPIRIT 2013 statement: defining standard protocol items for clinical trials. *Ann Intern Med* 2013;158(3):200-7. doi: 10.7326/0003-4819-158-3-201302050-00583 [published Online First: 2013/01/09]
27. Schulz KF, Altman DG, Moher D, et al. CONSORT 2010 Statement: updated guidelines for reporting parallel group randomised trials. *BMC Medicine* 2010;8(1):18. doi: 10.1186/1741-7015-8-18
28. Ciani O, Grigore B, Blommestein H, et al. Validity of Surrogate Endpoints and Their Impact on Coverage Recommendations: A Retrospective Analysis across International Health Technology Assessment Agencies. *Med Decis Making* 2021;41(4):439-52. doi: 10.1177/0272989x21994553 [published Online First: 2021/03/16]
29. FDA-NIH Biomarker Working Group. BEST (Biomarkers, endpoints, and other tools) resource [Internet]. 2021
30. Fleming TR, Powers JH. Biomarkers and surrogate endpoints in clinical trials. *Statistics in Medicine* 2012;31(25):2973-84. doi: https://doi.org/10.1002/sim.5403
31. Buyse M, Burzykowski T, Michiels S, et al. Individual- and trial-level surrogacy in colorectal cancer. *Stat Methods Med Res* 2008;17(5):467-75. doi: 10.1177/0962280207081864 [published Online First: 2008/02/21]
32. FDA. Table of Surrogate Endpoints That Were the Basis of Drug Approval or Licensure 2022 [Available from: https://www.fda.gov/drugs/development-resources/table-surrogate-endpoints-were-basis-drug-approval-or-licensure accessed 20/07/2022.
33. Moher D, Schulz KF, Simera I, et al. Guidance for developers of health research reporting guidelines. *PLoS Med* 2010;7(2):e1000217. doi: 10.1371/journal.pmed.1000217 [published Online First: 2010/02/20]
34. Munn Z, Peters MDJ, Stern C, et al. Systematic review or scoping review? Guidance for authors when choosing between a systematic or scoping review approach. *BMC Medical Research Methodology* 2018;18(1):143. doi: 10.1186/s12874-018-0611-x
35. Arksey H, O'Malley L. Scoping studies: towards a methodological framework. *International Journal of Social Research Methodology* 2005;8(1):19-32. doi: 10.1080/1364557032000119616
36. Levac D, Colquhoun H, O'Brien KK. Scoping studies: advancing the methodology. *Implementation Science* 2010;5(1):69. doi: 10.1186/1748-5908-5-69
37. Peters MDJ, Godfrey CM, Khalil H, et al. Guidance for conducting systematic scoping reviews. *JBI Evidence Implementation* 2015;13(3)
38. Fleming TR, DeMets DL. Surrogate end points in clinical trials: are we being misled? *Ann Intern Med* 1996;125(7):605-13. doi: 10.7326/0003-4819-125-7-199610010-00011
39. Kemp R, Prasad V. Surrogate endpoints in oncology: when are they acceptable for regulatory and clinical decisions, and are they currently overused? *BMC medicine* 2017;15(1):1-7.

For peer review only - http://bmjopen.bmj.com/site/about/guidelines.xhtml

40. Godin K, Stapleton J, Kirkpatrick SI, et al. Applying systematic review search methods to the grey literature: a case study examining guidelines for school-based breakfast programs in Canada. *Systematic reviews* 2015;4:138-38. doi: 10.1186/s13643-015-0125-0
41. Butcher NJ, Mew EJ, Monsour A, et al. Outcome reporting recommendations for clinical trial protocols and reports: a scoping review. *Trials* 2020;21(1):620. doi: 10.1186/s13063-020-04440-w
42. Covidence. Covidence systematic review software, Veritas Health Innovation, Melbourne, Australia.
43. Braun V, Clarke V. Using thematic analysis in psychology. *Qualitative research in psychology* 2006;3(2):77-101.
44. McGowan J, Straus S, Moher D, et al. Reporting scoping reviews-PRISMA ScR extension. *J Clin Epidemiol* 2020;123:177-79. doi: 10.1016/j.jclinepi.2020.03.016 [published Online First: 2020/04/02]

For peer review only

For peer review only - http://bmjopen.bmj.com/site/about/guidelines.xhtml

Supplementary File 1

Supplementary Table 1: Search strategy for MEDLINE

1	*Endpoint Determination/mt, st [Methods, Standards]
2	(Surrogate adj2 (measure* or outcome* or endpoint* or end point*)).ab,kw,ti.
3	(Surrogate and (controlled or trials)).ti.
4	(Endpoint adj1 determination).ab,kw,ti.
5	(Intermediate adj2 (outcome* or endpoint* or end point*)).ab,kw,ti.
6	1 or 2 or 3 or 4 or 5
7	Guideline Adherence/
8	Practice Guidelines as Topic/
9	Guidelines as Topic/
10	Checklist/
11	(Comparison or Regulation or regulatory or Policy or Decisions or Recommendation or Decision making or limitation* or understanding or reporting or critique or concept or conceptual or Validation or validity or recommendation or recommendations or guidance or advice or guideline* or guide line* or checklist or checklists or check list* or standard or standards or requirement* or instruction*).ti.
12	7 or 8 or 9 or 10 or 11
13	6 and 12
14	Clinical Trials as Topic/
15	Cohort Studies/
16	Randomized Controlled Trials as Topic/
17	"Reproducibility of Results"/
18	Research Design/
19	Data Collection/
20	Drug Approval/
21	Treatment Outcome/
22	Outcome Assessment, Health Care/
23	(outcomes or regulation).ti.
24	(clinical adj1 outcome assessment).ti.
25	clinical trials.ti.
26	14 or 15 or 16 or 17 or 18 or 19 or 20 or 21 or 22 or 23 or 24
27	13 and 26

For peer review only

For peer review only - http://bmjopen.bmj.com/site/about/guidelines.xhtml

Supplementary Table 2: Search strategy for EMBASE

1	(Surrogate adj2 (measure* or outcome* or endpoint* or end point*)).ab,kw,ti.
2	(Surrogate and (controlled or trials)).ti.
3	(Endpoint adj1 determination).ab,kw,ti.
4	(Intermediate adj2 (outcome* or endpoint* or end point*)).ab,kw,ti.
5	1 or 2 or 3 or 4
6	protocol compliance/
7	practice guideline/
8	checklist/
9	(Comparison or Regulation or regulatory or Policy or Decisions or Recommendation or Decision making or limitation* or understanding or reporting or critique or concept or conceptual or Validation or validity or recommendation or recommendations or guidance or advice or guideline* or guide line* or checklist or checklists or check list* or standard or standards or requirement* or instruction*).ti.
10	6 or 7 or 8 or 9
11	5 and 10
12	"clinical trial (topic)"/
13	cohort analysis/
14	"randomized controlled trial (topic)"/
15	reproducibility/
16	methodology/
17	information processing/
18	drug approval/
19	treatment outcome/
20	outcome assessment/
21	(outcomes or regulation).ti.
22	(clinical adj1 outcome assessment).ti.
23	clinical trials.ti.
24	12 or 13 or 14 or 15 or 16 or 17 or 18 or 19 or 20 or 21 or 22 or 23
25	11 and 24

For peer review only

For peer review only - http://bmjopen.bmj.com/site/about/guidelines.xhtml

Supplementary Table 3: Search strategy for Grey literature

Search strategy for grey literature. For each unique search, 100 hits will be reviewed for eligibility.

Source	Search strategy
**Google search engine**	Advanced search option (www.google.co.uk/advanced_search) will be used. Searches will combine terms appearing in the titles of for example:<br>• "surrogate endpoints" AND "recommendation" OR "guidance" OR "considerations"<br>• "clinical endpoints" AND "recommendation" OR "guidance" OR "considerations"
**Examples of relevant websites**	
**FDA (www.fda.gov)**	Use a search function to do a broad search using terms such as "surrogate endpoints", "clinical endpoints"
**MHRA (www.gov.uk/government/organisations/medicines-and-healthcare-products-regulatory-agency)**	No search option website will be browsed.
**European Medicines Agency (www.ema.europe.eu)**	Given its a large website, Advance Google search will be used, and search limited by the search by URL
**COMET initiative (www.comet-initiative.org)**	Searches done by options provided by the website. For example: by Method= Literature review; by Study Type =Commentary, COS methods research

For peer review only

For peer review only - http://bmjopen.bmj.com/site/about/guidelines.xhtml

Protocol for scoping and targeted reviews to support development of SPIRIT and CONSORT extensions for randomised controlled trials with surrogate primary endpoints

Anthony Muchai Manyara[1], Philippa Davies[2], Derek Stewart[3], Valerie Wells[1], Christopher J Weir[4], Amber Young[2], Rod S Taylor[1,4], Oriana Ciani[5]

1 MRC/CSO Social and Public Health Sciences Unit, Institute of Health and Wellbeing, University of Glasgow, UK

2 Population Health Sciences, Bristol Medical School, University of Bristol, UK

3 Patient and Public Involvement lead, UK

4 Edinburgh Clinical Trials Unit, Usher Institute, University of Edinburgh, UK

5 Robertson Centre for Biostatistics, Institute of Health and Well Being, University of Glasgow, UK

6 SDA Bocconi School of Management, Milan, Italy

Correspondence to Anthony Manyara; Anthony.Manyara@glasgow.ac.uk

For peer review only - http://bmjopen.bmj.com/site/about/guidelines.xhtml

Abstract

Introduction

Using a surrogate endpoint as a substitute for a primary patient-relevant outcome enables randomised controlled trials (RCTs) to be conducted more efficiently, i.e., with shorter time, smaller sample size, and lower cost. However, there is currently no consensus-driven guideline for the reporting of RCTs using a surrogate endpoint as a primary outcome; therefore, we seek to develop SPIRIT (Standard Protocol Items: Recommendations for Interventional Trials) and CONSORT (Consolidated Standards of Reporting Trials) extensions to improve the design and reporting of these trials. As an initial step, scoping and targeted reviews will identify potential items for inclusion in the extensions and participants to contribute to a Delphi consensus process.

Methods and analysis

The scoping review will search and include literature reporting on the current understanding, limitations, and guidance on using surrogate endpoints in trials. Relevant literature will be identified through: 1) bibliographic databases; 2) grey literature; 3) handsearching of reference lists; and 4) solicitation from experts. Data from eligible records will be thematically analysed into potential items for inclusion in extensions. The targeted review will search for RCT reports and protocols published from 2017-2021 in six high impact general medical journals. Trial corresponding author contacts will be listed as potential participants for the Delphi exercise.

Ethics and dissemination

Ethical approval is not required. The reviews will support the development of SPIRIT and CONSORT extensions for reporting surrogate primary endpoints (.surrogate endpoint as the primary outcome) The findings will be published in open-access publications.

This review has been prospectively registered in the OSF Registration DOI: 10.17605/OSF.IO/WP3QH.

Keywords: Surrogate endpoints, randomised controlled trials, Reporting guidelines

For peer review only - http://bmjopen.bmj.com/site/about/guidelines.xhtml

Strengths and limitations of this study

- Our scoping review will use rigorous methods to identify literature using multiple sources with no restriction to regions or time periods.
- The targeted review will identify recent randomised controlled trials that have used surrogate primary endpoints from six high impact journals.
- Due to lack of resources for translation, we will only include records in English or Italian.
- Using a purposively selected set of journals for the targeted review means that our review of recent RCT protocols and trials is not exhaustive and may lack generalisability.

For peer review only

For peer review only - http://bmjopen.bmj.com/site/about/guidelines.xhtml

Introduction

Randomised controlled trials (RCTs), that are well designed, conducted, and reported, provide rigorous scientific evidence for evaluating the effectiveness and safety of interventions intended to impact health [1 2]. Nevertheless, to meet the scientific, ethical, and regulatory requirements, the conduct and delivery of RCTs is becoming increasingly resource and time-intensive [3], with median cost estimates of up to US$ 21.4 million for phase three trials [4]. The use of a surrogate endpoint as a substitute for a primary final patient relevant outcome [5] provides a potentially attractive solution for improving efficiency of RCTs, i.e., shorter follow up, smaller sample size, and, as a result, lower cost.

A key rationale for the use of a surrogate endpoint is that the intervention effect on the surrogate fully captures the intervention effect on the final patient relevant outcome [6]. Consideration of surrogate endpoints in RCTs has traditionally focused on the regulatory setting for pharmaceuticals and whether biomarkers are "likely to predict" patient-centred outcomes of interest (e.g., systolic blood pressure for stroke, low density lipoprotein cholesterol for myocardial infarction, and HIV viral load for development of AIDS). However, it is important to acknowledge a more wider application in RCTs of intermediate outcomes that are believed to capture the causal pathway through which pharmaceutical, surgical, organizational or public health interventions impact the ultimate patient-relevant outcome (e.g., hospice enrolment for mortality with an intervention aimed at improving end of life care [7]; fruit and vegetable consumption for cardiovascular events for a behavioural intervention designed to improve cardiovascular risk [8]). To be regarded as a valid surrogate endpoint, a biomarker or intermediate outcome is required: 1) to reliably predict the PRFO in individual trial participants ('individual level' or 'patient-level' surrogacy); and 2) the intervention effect on the surrogate endpoint should reliably predict the intervention effect on the PRFO ('trial-level' surrogacy) based on evidence from meta-analyses of RCT data on both outcomes[9 10]. Statistical surrogate validation uses various statistical methods, including meta-analyses of RCT aggregate and/or individual patient data[11 12], principal stratification [13], causal inference [14 15], bivariate network meta-analysis methods[16 17] and information theory [18]. However, surrogate validation should extend beyond statistical validity to include a multifaceted approach comprising of biological plausibility rationale and "face validity" of the surrogates in trials[19].

For peer review only - http://bmjopen.bmj.com/site/about/guidelines.xhtml

Despite the potential appeal of surrogate endpoints in RCTs, their use in clinical and policy decision making remains controversial. An empirical analysis has found that RCTs using a surrogate endpoint primary outcome typically report 46% larger treatment effects compared to RCTs with final patient relevant primary outcomes [20]. This finding is supported by theoretical analyses [21]. Concerningly, some approvals based on surrogate endpoints have led to the 'real world' use of interventions that fail to demonstrate their predicted benefit(s) on the ultimate patient-centred outcome of interest and even more worryingly, that result in more harm than good [22 23]. Therefore, design and reporting of RCTs using surrogate endpoints should clearly convey the uncertainty and risks associated with their use. However, audits of RCTs to date have found this not to be the case. An analysis of 626 RCTs published in 2005 and 2006 found that 107 (17%) used a surrogate primary endpoint (surrogate endpoint as a the primary outcome) and of these, only a third discussed whether the surrogate was a valid predictor of patient-relevant outcomes [24]. Furthermore, a review of 220 cardiovascular surrogate trials found that only 59 (27%) had evidence validating the benefits of interventions on a final patient-relevant outcome [25].

Reporting guidelines can guide design and improve the reporting of RCTs at both the protocol and report stages. Two established guidelines are: SPIRIT (Standard Protocol Items: Recommendations for Interventional Trials) 2013 statement: a 33-item checklist used to guide the drafting of RCT protocols [26]; and CONSORT (Consolidated Standards of Reporting Trials) 2010 Statement is a 25-item checklist used to improve reporting of conducted trials [27]. Yet, although SPIRIT and CONSORT (and related extensions) provide general guidance on outcome reporting, there remains no standard evidence-based reference for dealing with surrogacy of the primary endpoint. Improving transparency in the reporting of trials using surrogates would enable the evidence base for the surrogate to be more effectively scrutinised. Therefore, we aim to develop extensions to report trial protocols and reports that use surrogate primary endpoints: SPIRIT-SURROGATE and CONSORT-SURROGATE, respectively. The extensions focus on trials using surrogate endpoints as primary outcomes (including as part of a composite outcome) as these would inform trial conclusions and interpretations of results and possible approval of ~~Our~~interventions. Our working definition of a surrogate endpoint is: 'a biomarker or intermediate outcome used to substitute and predict for a final patient relevant outcome (i.e., characteristic or variable that captures how a patient feels, functions, or how long they survive, such as the outcomes of mortality or health-related quality of life)' [5 6 28]. Additionally, reference of surrogate endpoints in this

For peer review only - http://bmjopen.bmj.com/site/about/guidelines.xhtml

project refers to statistically validated surrogate endpoints (e.g., change in systolic blood pressure for cardiovascular mortality in anti-hypertensive treatments[29,30], disease-free survival (and progression free survival in advanced disease) in colorectal cancer[31]) and non-validated surrogates for which are 'reasonably likely to predict health benefit' (e.g., reduction in amyloid load in Alzheimer's disease)[29,32] ~~[29,32,33]~~

To develop these extensions, we will closely follow the EQUATOR (Enhancing the QUAlity and Transparency Of health Research) network's recommended steps for developing a health research reporting guideline [34]. We have structured our project into four phases: Phase 1 (Literature reviews), Phase 2 (Delphi study), Phase 3 (Consensus meeting), and Phase 4 (Knowledge translation). This protocol outlines the activities and procedures of Phase 1 consisting of scoping and targeted reviews. The scoping review will be used to: synthesise current evidence and guidance on using surrogate endpoints to generate candidate items for potential inclusion in extensions; and identify surrogate content experts for recruitment in the Delphi study (Phase 2). The primary aim of the targeted review is to identify trial investigators who have led an RCT using a surrogate endpoint to be invited to participate in the Delphi exercise. A secondary aim will be to archive identified protocols and trials and use them as a 'baseline' for future evaluation of the impact of developed extensions on the reporting practice of future RCT protocols and reports.

For peer review only

Methods and analysis

Scoping review

The scoping review was considered to be the most suitable knowledge synthesis approach for addressing the broad aim of this study [35]. The scoping review will be conducted using a methodological framework proposed by Arksey and O'Malley [36], and enhancements proposed to this framework by Levac et al [37] and Peters et al [38]. This will involve six stages: formulating a research question; identifying relevant studies; inclusion of studies; charting data; summarising and reporting results; and consultation [36].

Framework stage one: Formulating the research question

This scoping review seeks to identify a list of items that should be considered when reporting RCT protocols and reports which use surrogate endpoints. Therefore, our overarching research question combines a broad scope and a specific area of inquiry [37] (i.e., surrogate endpoint use): what is the current understanding, advice, and guidance on using surrogate endpoints in RCTs? Specific research questions are:

For peer review only - http://bmjopen.bmj.com/site/about/guidelines.xhtml

1. How are surrogate endpoints defined?
2. What are the limitations of using surrogate endpoints in RCTs?
3. When is the use of surrogate endpoints acceptable?
4. What published advice and guidance exists on reporting RCTs protocols and reports using surrogate endpoints?

There is a possibility of modification of these research questions during the literature reviewing and this will be reported when publishing the findings.

Framework stage two: Identifying relevant literature

We will adopt a search approach that balances comprehensiveness, breadth, and feasibility [37]. Relevant literature will be identified through: 1) electronic bibliographic databases (Excerpta Medica Database (EMBASE), Medical Literature Analysis and Retrieval System Online (MEDLINE), Cochrane Methodology Register); 2) Grey literature (Google and relevant website search); 3) handsearching of reference lists; and 4) solicitation for additional literature from expert colleagues [36-38].

Electronic databases and grey literature search will be supported by an experienced information specialist (VW). We have developed an initial search strategy for MEDLINE and EMBASE which combines "surrogate endpoints", "guidelines", and "trials" related search terms (see Table 1 and 2 in Supplementary File 1). This strategy was checked for validity against four highly cited articles (>50 citations) that answer either of our specific research questions [20 24 39 40].

For grey literature, search strategies will be modified for each of the websites and for each strategy, the search terms and the number of results retrieved and/or screened will be recorded [41]. Table 3 in Supplementary File 1 shows search strategies to be used in the Google search engine and in some of the relevant websites. Generally, the strategies will include combination of search terms (e.g., "surrogate endpoints" AND "guidance") in Google advanced search; broad searches (e.g., surrogate endpoints) using the website search function; and browsing for websites without a search function. For large websites (e.g., www.ema.europe.eu), Google advanced search will be used, and search limited to the website URL. The first 100 hits in each search will be screened for eligibility to balance between feasibility and relevancy of records [41]. One reviewer will screen searches on the Google search engine or websites using title and, if present, any short text underneath.

For peer review only - http://bmjopen.bmj.com/site/about/guidelines.xhtml

All reference lists of included full texts will be screened to identify relevant records. We will solicit for additional resources from surrogate and outcome measurement experts including authors of a recent scoping review (on "outcome reporting recommendations for trial protocols and reports") which identified eight documents that focused on reporting recommendations for surrogate outcomes [42].

Framework stage three: Literature selection

Databases search results will be exported to Endnote version X9 for the removal of duplicates. The remaining records will be exported to Covidence [43] for eligibility screening based on title, abstract, and full-text reading by two reviewers. Title and abstract screening of grey literature will be done in respective websites by one reviewer and full-text screening done from HTML files by two reviewers.

Once full-text screening has been concluded, reviewers will hand search reference lists of all included full-texts for relevant records. The identified records combined with those supplied from experts will undergo full-text screening. Records will be eligible for inclusion if they report findings relevant to any research question. While we will mainly include records that are peer-reviewed literature, academic or regulatory grey literature (e.g., white papers), reviewers will make judgements on inclusion of other records (e.g., conference abstracts) based on relevance to review questions and trustworthiness of evidence presented. We will not restrict our inclusion of literature to regions or time periods. However, we will only include records in English or Italian due to lack of resources for translation. Disagreements between reviewers will be resolved by consensus or, if necessary, involving a third reviewer.

Framework stage four: Charting the findings

The following data will be extracted: author (and contact of corresponding author), publication year, country, author affiliation category (e.g., academic, regulatory body, patient/public forums), record type (e.g., review article, commentary, regulatory guidance), research area if specified, funding if stated, and findings relevant to research questions (i.e., definition, limitations, acceptability, guidance on surrogate endpoints use). A pilot will be undertaken to check if the data extraction template needs modification. All data extraction will be done by one reviewer. At the start of extraction, a subset of extracted data (~10% of records) will be checked for accuracy by a second reviewer and if accurate the first reviewer will proceed to extract in all other records.

For peer review only - http://bmjopen.bmj.com/site/about/guidelines.xhtml

Framework stage five: Synthesis and reporting the findings

All analysis will be done in Microsoft Excel. Descriptive data (i.e., publication year, country/region, author affiliation category, record type) will be analysed using counts and percentages and presented in tables, graphs or as text. Data related to research questions (e.g., key messages/advice/guidelines on surrogate endpoints use) will be collated verbatim under each research question. A simple form of thematic analysis [44] will then be used to synthesise data. Two reviewers will independently read the collated data under each research question and for each record, summarise it into: 1) item(s) to be considered when reporting protocols and trials using surrogate endpoints; and 2) whether the items are new or modifications to the SPIRIT and/or CONSORT checklist items and for new items, the section of the checklist where they should be reported. The reviewers will then meet for a virtual workshop to discuss and agree on items and their designated sections of the checklist. We will report the findings in an open-access peer reviewed publication using the Preferred Reporting Items for Systematic reviews and Meta-Analysis for Scoping Reviews (PRISMA-ScR) [45].

Framework stage six: Consultation exercise

The aim of consultation is to share scoping review findings with stakeholders so as to identify additional relevant resources and valuable insights that the scoping review findings may have missed [36]. Nevertheless, it is important to specify when, how, and why to do consultation, the types of stakeholder involved, and how to integrate the information with review findings [37]. We will use preliminary review findings to seek insights, through virtual meetings, from Public and Patient Involvement (PPI) representatives on the identified items for reporting surrogate endpoints. Our project PPI Lead (DS) will coordinate consultation with PPI representatives, and this will offer an opportunity for knowledge transfer and exchange. Additionally, we will invite our multidisciplinary expert advisory Executive Committee members (see Acknowledgement) and the MRC-NIHR Trials Methodology Research Partnership Outcomes Working Group (www.methodologyhubs.mrc.ac.uk/), specifically the Surrogate Outcomes subgroup, to comment on any additional resources, items, and perspectives not included in the preliminary findings. Review comments on the preliminary findings document or detailed notes taken during consultation meetings will be used in summarising and integrating suggested items into the review findings.

Targeted review

The targeted reviews are intended to identify trial investigators who have led an RCT assessing a surrogate endpoint and protocols and trials that have a primary surrogate endpoint

For peer review only - http://bmjopen.bmj.com/site/about/guidelines.xhtml

[20]. MEDLINE through PubMed will be searched for RCTs published in the last five years (2017-2021) in six high impact general medical journals: *Annals of Internal Medicine*, *BMJ*, *Journal of the American Medical Association*, *New England Journal of Medicine*, *Lancet*, and *PLoS Medicine.* Use of general medical journals allows for inclusion of records across a range of clinical areas. Given the focus of the project on reporting guidelines for trial protocols, we also will search two journals widely used for publishing RCT protocols: *BMJ Open* and *Trials*. We will include trial protocols and reports that use outcomes that meet our working definition of surrogate endpoints.

All identified protocols and trials will be exported to Endnote version X9 for the removal of duplicates and exported to Covidence [43] for eligibility screening. Given the primary objective of this review is to identify trial investigators who have used surrogate endpoints, screening will be limited to titles and abstracts. Two reviewers will screen all records and include those protocols and randomised trials that use surrogate primary endpoints and report intervention studies. A more in-depth screening and analysis of the full texts will be done as part of an upcoming project, acting as a baseline to evaluate the impact of the extensions (post-publication) on the reporting of RCT protocols and trials.

From the included records, one reviewer will extract the title, journal, year of publication, research area, corresponding author name, institutional affiliation, and email address. These data will be used to sample and recruit participants for the Delphi study (Phase 2 of the project).

Patient and public involvement

One of the project team members (DS) is a leading PPI advocate who has been involved in health research at local, national, and international level. As outlined, PPI will be integrated in stage six of the scoping review. We are additionally exploring how patients and the public can be meaningfully involved in this project.

Limitations

Although we will use four strategies in our scoping review searches, our inclusion will be limited to records in English and Italian language hence exclusion of non-English/Italian literature. Nevertheless, our review does not aim to be exhaustive but to identify important items for consideration when using surrogate endpoints and it is highly likely items synthesised from records in the English and Italian language would be transferable to other

For peer review only - http://bmjopen.bmj.com/site/about/guidelines.xhtml

settings. Using an approach of a purposively selected set of journals means our targeted review of recent RCT protocols and trials is not exhaustive and may lack generalisability.

Conclusion

This protocol has described the procedures to be followed in conducting a scoping review and targeted review to support development of SPIRIT and CONSORT extensions of RCTs reporting primary outcomes that are surrogate endpoints. Use of scoping review methodology to identify candidate items to be rated by experts through a Delphi methodology [46] is consistent with EQUATOR guidelines [34] and has been used in recent developed extensions including CONSORT-ROUTINE [47] and Adaptive designs CONSORT Extension (ACE) [48]. Our targeted review will provide a 'baseline' of current RCT reporting that can be used to assess the impact of our developed extensions on future RCTs. The SPIRIT-SURROGATE and CONSORT-SURROGATE extensions seek to improve transparency of reporting and design of RCTs that use surrogate endpoints and thereby contribute to better clinical and policy decision-making, and ultimately health of the population.

Ethics and dissemination

The reviews do not require ethics approval. The reviews findings will be disseminated through conference presentations and open-access publications.

Author contributionsContributorship Statement

PD, CJW, AY, RT, and OC were involved in funding acquisition. AMM, PD, DS, CJW, AY, RT, and OC were involved in the initial phases of study conception and design. AMM, VW, RT, and OC were involved in design of the search strategy and responsible for the first draft of the manuscript. PD, DS, CJW, and AY reviewed the first draft and all authors approved the final version.

Competing Interests

None to declare.

For peer review only - http://bmjopen.bmj.com/site/about/guidelines.xhtml

Funding

The development of SPIRIT and CONSORT extensions has been funded by the Medical Research Council (PD, CJW, AY, RT, OC, grant number MR/V038400/1).

Acknowledgments

We acknowledge our advisory Executive Committee members who will oversight the project and contribute to the consultation stage of the scoping review: Professor Joseph Ross (Chair of the Executive Committee); Professor Martin Offringa; Dr. Nancy Butcher; Professor An-Wen Chan; Professor Gary Collins; Professor Sylwia Bujkiewicz; Dr Dalia Dawoud; and Dr Mario Ouwens.

References

1. Akobeng AK. Understanding randomised controlled trials. *Archives of Disease in Childhood* 2005;90(8):840-44. doi: 10.1136/adc.2004.058222
2. Hariton E, Locascio JJ. Randomised controlled trials - the gold standard for effectiveness research: Study design: randomised controlled trials. *BJOG* 2018;125(13):1716-16. doi: 10.1111/1471-0528.15199 [published Online First: 2018/06/19]
3. Umscheid CA, Margolis DJ, Grossman CE. Key concepts of clinical trials: a narrative review. *Postgraduate medicine* 2011;123(5):194-204. doi: 10.3810/pgm.2011.09.2475
4. Martin L, Hutchens M, Hawkins C, et al. How much do clinical trials cost? *Nature Reviews Drug Discovery* 2017;16(6):381-82. doi: 10.1038/nrd.2017.70
5. Biomarkers Definitions Working Group. Biomarkers and surrogate endpoints: Preferred definitions and conceptual framework. *Clinical Pharmacology & Therapeutics* 2001;69(3):89-95. doi: https://doi.org/10.1067/mcp.2001.113989
6. DeMets DL, Psaty BM, Fleming TR. When Can Intermediate Outcomes Be Used as Surrogate Outcomes? *Jama* 2020;323(12):1184-85. doi: 10.1001/jama.2020.1176 [published Online First: 2020/02/28]
7. Casarett D, Karlawish J, Morales K, et al. Improving the use of hospice services in nursing homes: a randomized controlled trial. *Jama* 2005;294(2):211-7. doi: 10.1001/jama.294.2.211 [published Online First: 2005/07/15]
8. Domke A, Keller J, Heuse S, et al. Immediate effects of a very brief planning intervention on fruit and vegetable consumption: A randomized controlled trial. *Applied Psychology: Health and Well-Being* 2021;13(2):377-93. doi: https://doi.org/10.1111/aphw.12254
9. Buyse M, Molenberghs G, Burzykowski T, et al. The validation of surrogate endpoints in meta-analyses of randomized experiments. *Biostatistics* 2000;1(1):49-67. doi: 10.1093/biostatistics/1.1.49 [published Online First: 2003/08/23]
10. Buyse M, Saad ED, Burzykowski T, et al. Surrogacy Beyond Prognosis: The Importance of "Trial-Level" Surrogacy. *The Oncologist* 2022;27(4):266-71. doi: 10.1093/oncolo/oyac006
11. Xie W, Halabi S, Tierney JF, et al. A Systematic Review and Recommendation for Reporting of Surrogate Endpoint Evaluation Using Meta-analyses. *JNCI Cancer Spectr* 2019;3(1):pkz002. doi: 10.1093/jncics/pkz002 [published Online First: 2019/07/31]

For peer review only - http://bmjopen.bmj.com/site/about/guidelines.xhtml

12. Daniels MJ, Hughes MD. Meta-analysis for the evaluation of potential surrogate markers. *Stat Med* 1997;16(17):1965-82. doi: 10.1002/(sici)1097-0258(19970915)16:17<1965::aid-sim630>3.0.co;2-m [published Online First: 1997/09/26]
13. Weir CJ, Walley RJ. Statistical evaluation of biomarkers as surrogate endpoints: a literature review. *Stat Med* 2006;25(2):183-203. doi: 10.1002/sim.2319
14. Alonso A, Van der Elst W, Molenberghs G, et al. On the relationship between the causal-inference and meta-analytic paradigms for the validation of surrogate endpoints. *Biometrics* 2015;71(1):15-24. doi: 10.1111/biom.12245 [published Online First: 2014/10/03]
15. Vanderweele TJ. Surrogate measures and consistent surrogates. *Biometrics* 2013;69(3):561-9. doi: 10.1111/biom.12071 [published Online First: 2013/10/01]
16. Bujkiewicz S, Jackson D, Thompson JR, et al. Bivariate network meta-analysis for surrogate endpoint evaluation. *Stat Med* 2019;38(18):3322-41. doi: 10.1002/sim.8187 [published Online First: 2019/05/28]
17. Papanikos T, Thompson JR, Abrams KR, et al. Bayesian hierarchical meta-analytic methods for modeling surrogate relationships that vary across treatment classes using aggregate data. *Stat Med* 2020;39(8):1103-24. doi: 10.1002/sim.8465 [published Online First: 2020/01/29]
18. Alonso A, Van der Elst W, Molenberghs G, et al. An information-theoretic approach for the evaluation of surrogate endpoints based on causal inference. *Biometrics* 2016;72(3):669-77. doi: 10.1111/biom.12483 [published Online First: 2016/02/13]
19. Green E, Yothers G, Sargent DJ. Surrogate endpoint validation: statistical elegance versus clinical relevance. *Statistical Methods in Medical Research* 2008;17(5):477-86. doi: 10.1177/0962280207081863
20. Ciani O, Buyse M, Garside R, et al. Comparison of treatment effect sizes associated with surrogate and final patient relevant outcomes in randomised controlled trials: meta-epidemiological study. *BMJ : British Medical Journal* 2013;346:f457. doi: 10.1136/bmj.f457
21. Walter SD, Sun X, Heels-Ansdell D, et al. Treatment effects on patient-important outcomes can be small, even with large effects on surrogate markers. *J Clin Epidemiol* 2012;65(9):940-5. doi: 10.1016/j.jclinepi.2012.02.012 [published Online First: 2012/05/23]
22. Grimes DA, Schulz KF. Surrogate end points in clinical research: hazardous to your health. *Obstet Gynecol* 2005;105(5 Pt 1):1114-8. doi: 10.1097/01.aog.0000157445.67309.19 [published Online First: 2005/05/03]
23. Thomas R. Fleming, David L. DeMets. Surrogate End Points in Clinical Trials: Are We Being Misled? *Annals of Internal Medicine* 1996;125(7):605-13. doi: 10.7326/0003-4819-125-7-199610010-00011 %m 8815760
24. la Cour JL, Brok J, Gøtzsche PC. Inconsistent reporting of surrogate outcomes in randomised clinical trials: cohort study. *BMJ* 2010;341:c3653-c53. doi: 10.1136/bmj.c3653
25. Bikdeli B, Punnanithinont N, Akram Y, et al. Two Decades of Cardiovascular Trials With Primary Surrogate Endpoints: 1990-2011. *J Am Heart Assoc* 2017;6(3):e005285. doi: 10.1161/JAHA.116.005285
26. Chan AW, Tetzlaff JM, Altman DG, et al. SPIRIT 2013 statement: defining standard protocol items for clinical trials. *Ann Intern Med* 2013;158(3):200-7. doi: 10.7326/0003-4819-158-3-201302050-00583 [published Online First: 2013/01/09]
27. Schulz KF, Altman DG, Moher D, et al. CONSORT 2010 Statement: updated guidelines for reporting parallel group randomised trials. *BMC Medicine* 2010;8(1):18. doi: 10.1186/1741-7015-8-18
28. Ciani O, Grigore B, Blommestein H, et al. Validity of Surrogate Endpoints and Their Impact on Coverage Recommendations: A Retrospective Analysis across International Health Technology Assessment Agencies. *Med Decis Making* 2021;41(4):439-52. doi: 10.1177/0272989x21994553 [published Online First: 2021/03/16]
29. FDA-NIH Biomarker Working Group. BEST (Biomarkers, endpoints, and other tools) resource [Internet]. 2021

For peer review only

For peer review only - http://bmjopen.bmj.com/site/about/guidelines.xhtml

30. Fleming TR, Powers JH. Biomarkers and surrogate endpoints in clinical trials. *Statistics in Medicine* 2012;31(25):2973-84. doi: https://doi.org/10.1002/sim.5403
31. Buyse M, Burzykowski T, Michiels S, et al. Individual- and trial-level surrogacy in colorectal cancer. *Stat Methods Med Res* 2008;17(5):467-75. doi: 10.1177/0962280207081864 [published Online First: 2008/02/21]
32. FDA. Table of Surrogate Endpoints That Were the Basis of Drug Approval or Licensure 2022 [Available from: https://www.fda.gov/drugs/development-resources/table-surrogate-endpoints-were-basis-drug-approval-or-licensure accessed 20/07/2022.
33. Fleming TR, Powers JH. Biomarkers and surrogate endpoints in clinical trials. *Stat Med* 2012;31(25):2973-84. doi: 10.1002/sim.5403 [published Online First: 2012/06/20]
34. Moher D, Schulz KF, Simera I, et al. Guidance for developers of health research reporting guidelines. *PLoS Med* 2010;7(2):e1000217. doi: 10.1371/journal.pmed.1000217 [published Online First: 2010/02/20]
35. Munn Z, Peters MDJ, Stern C, et al. Systematic review or scoping review? Guidance for authors when choosing between a systematic or scoping review approach. *BMC Medical Research Methodology* 2018;18(1):143. doi: 10.1186/s12874-018-0611-x
36. Arksey H, O'Malley L. Scoping studies: towards a methodological framework. *International Journal of Social Research Methodology* 2005;8(1):19-32. doi: 10.1080/1364557032000119616
37. Levac D, Colquhoun H, O'Brien KK. Scoping studies: advancing the methodology. *Implementation Science* 2010;5(1):69. doi: 10.1186/1748-5908-5-69
38. Peters MDJ, Godfrey CM, Khalil H, et al. Guidance for conducting systematic scoping reviews. *JBI Evidence Implementation* 2015;13(3)
39. Fleming TR, DeMets DL. Surrogate end points in clinical trials: are we being misled? *Ann Intern Med* 1996;125(7):605-13. doi: 10.7326/0003-4819-125-7-199610010-00011
40. Kemp R, Prasad V. Surrogate endpoints in oncology: when are they acceptable for regulatory and clinical decisions, and are they currently overused? *BMC medicine* 2017;15(1):1-7.
41. Godin K, Stapleton J, Kirkpatrick SI, et al. Applying systematic review search methods to the grey literature: a case study examining guidelines for school-based breakfast programs in Canada. *Systematic reviews* 2015;4:138-38. doi: 10.1186/s13643-015-0125-0
42. Butcher NJ, Mew EJ, Monsour A, et al. Outcome reporting recommendations for clinical trial protocols and reports: a scoping review. *Trials* 2020;21(1):620. doi: 10.1186/s13063-020-04440-w
43. Covidence. Covidence systematic review software, Veritas Health Innovation, Melbourne, Australia.
44. Braun V, Clarke V. Using thematic analysis in psychology. *Qualitative research in psychology* 2006;3(2):77-101.
45. McGowan J, Straus S, Moher D, et al. Reporting scoping reviews-PRISMA ScR extension. *J Clin Epidemiol* 2020;123:177-79. doi: 10.1016/j.jclinepi.2020.03.016 [published Online First: 2020/04/02]
46. Trevelyan EG, Robinson N. Delphi methodology in health research: how to do it? *European Journal of Integrative Medicine* 2015;7(4):423-28.
47. Kwakkenbos L, Imran M, McCord KA, et al. Protocol for a scoping review to support development of a CONSORT extension for randomised controlled trials using cohorts and routinely collected health data. *BMJ Open* 2018;8(8):e025266. doi: 10.1136/bmjopen-2018-025266
48. Dimairo M, Coates E, Pallmann P, et al. Development process of a consensus-driven CONSORT extension for randomised trials using an adaptive design. *BMC Medicine* 2018;16(1):210. doi: 10.1186/s12916-018-1196-2

For peer review only

For peer review only - http://bmjopen.bmj.com/site/about/guidelines.xhtml

~~Author contributions~~

~~PD, CJW, AY, RT, and OC were involved in funding acquisition. AMM, PD, DS, CJW, AY, RT, and OC were involved in the initial phases of study conception and design. AMM, VW, RT, and OC were involved in design of the search strategy and responsible for the first draft of the manuscript. PD, DS, CJW, and AY reviewed the first draft and all authors approved the final version.~~

For peer review only

For peer review only - http://bmjopen.bmj.com/site/about/guidelines.xhtml

Acknowledgments

We acknowledge our advisory Executive Committee members who will oversight the project and contribute to the consultation stage of the scoping review: Professor Joseph Ross (Chair of the Executive Committee); Professor Martin Offringa; Dr. Nancy Butcher; Professor An-Wen Chan; Professor Gary Collins; Professor Sylwia Bujkiewicz; Dr Dalia Dawoud; and Dr Mario Ouwens.

Funding Statement

The development of SPIRIT and CONSORT extensions has been funded by the Medical Research Council (PD, CJW, AY, RT, OC, grant number MR/V038400/1).

Competing Interest

None to declare.

Supplementary File 1

Search strategy for MEDLINE and Embase

Table 1: MEDLINE

1	*Endpoint Determination/mt, st [Methods, Standards]
2	(Surrogate adj2 (measure* or outcome* or endpoint* or end point*)).ab,kw,ti.
3	(Surrogate and (controlled or trials)).ti.
4	(Endpoint adj1 determination).ab,kw,ti.

For peer review only - http://bmjopen.bmj.com/site/about/guidelines.xhtml

5	(Intermediate adj2 (outcome* or endpoint* or end point*)).ab,kw,ti.
6	1 or 2 or 3 or 4 or 5
7	Guideline Adherence/
8	Practice Guidelines as Topic/
9	Guidelines as Topic/
10	Checklist/
11	(Comparison or Regulation or regulatory or Policy or Decisions or Recommendation or Decision making or limitation* or understanding or reporting or critique or concept or conceptual or Validation or validity or recommendation or recommendations or guidance or advice or guideline* or guide line* or checklist or checklists or check list* or standard or standards or requirement* or instruction*).ti.
12	7 or 8 or 9 or 10 or 11
13	6 and 12
14	Clinical Trials as Topic/
15	Cohort Studies/
16	Randomized Controlled Trials as Topic/
17	"Reproducibility of Results"/
18	Research Design/
19	Data Collection/
20	Drug Approval/

For peer review only - http://bmjopen.bmj.com/site/about/guidelines.xhtml

21	Treatment Outcome/
22	Outcome Assessment, Health Care/
23	(outcomes or regulation).ti.
24	(clinical adj1 outcome assessment).ti.
25	clinical trials.ti.
26	14 or 15 or 16 or 17 or 18 or 19 or 20 or 21 or 22 or 23 or 24
27	13 and 26

For peer review only

For peer review only - http://bmjopen.bmj.com/site/about/guidelines.xhtml

Table 2: EMBASE

1	(Surrogate adj2 (measure* or outcome* or endpoint* or end point*)).ab,kw,ti.
2	(Surrogate and (controlled or trials)).ti.
3	(Endpoint adj1 determination).ab,kw,ti.
4	(Intermediate adj2 (outcome* or endpoint* or end point*)).ab,kw,ti.
5	1 or 2 or 3 or 4
6	protocol compliance/
7	practice guideline/
8	checklist/
9	(Comparison or Regulation or regulatory or Policy or Decisions or Recommendation or Decision making or limitation* or understanding or reporting or critique or concept or conceptual or Validation or validity or recommendation or recommendations or guidance or advice or guideline* or guide line* or checklist or checklists or check list* or standard or standards or requirement* or instruction*).ti.
10	6 or 7 or 8 or 9
11	5 and 10
12	"clinical trial (topic)"/
13	cohort analysis/
14	"randomized controlled trial (topic)"/

For peer review only - http://bmjopen.bmj.com/site/about/guidelines.xhtml

~~15~~	~~reproducibility/~~
~~16~~	~~methodology/~~
~~17~~	~~information processing/~~
~~18~~	~~drug approval/~~
~~19~~	~~treatment outcome/~~
~~20~~	~~outcome assessment/~~
~~21~~	~~(outcomes or regulation).ti.~~
~~22~~	~~(clinical adj1 outcome assessment).ti.~~
~~23~~	~~clinical trials.ti.~~
~~24~~	~~12 or 13 or 14 or 15 or 16 or 17 or 18 or 19 or 20 or 21 or 22 or 23~~
~~25~~	~~11 and 24~~

For peer review only - http://bmjopen.bmj.com/site/about/guidelines.xhtml

Table 3: Grey literature

Search strategy for grey literature. For each unique search, 100 hits will be reviewed for eligibility.

Source	Search strategy
**Google search engine**	Advanced search option (**Error! Hyperlink reference not valid.**) will be used. Searches will combine terms appearing in the titles of for example:<br>• "surrogate endpoints" AND "recommendation" OR "guidance" OR "considerations"<br>• "clinical endpoints" AND "recommendation" OR "guidance" OR "considerations"
**Examples of relevant websites**	
**FDA**<br>**(Error! Hyperlink reference not valid.)**	Use a search function to do a broad search using terms such as "surrogate endpoints", "clinical endpoints"
**MHRA**	No search option website will be browsed.

For peer review only - http://bmjopen.bmj.com/site/about/guidelines.xhtml

~~(Error! Hyperlink reference not valid.)~~	
~~European Medicines Agency (Error! Hyperlink reference not valid.)~~	~~Given its a large website, Advance Google search will be used, and search limited by the search by URL~~
~~COMET initiative~~<br><br>~~(Error! Hyperlink reference not valid.)~~	~~Searches done by options provided by the website. For example: by Method= Literature review; by Study Type =Commentary, COS methods research~~

For peer review only - http://bmjopen.bmj.com/site/about/guidelines.xhtml

Protocol for scoping and targeted reviews to support development of SPIRIT and CONSORT extensions for randomised controlled trials with surrogate primary endpoints: Authors response to review feedback

Reviewer comments	Authors response	Modification to manuscript
**Comments from the Reviewer:**		
The paper was revised by authors. However, I could not see any serious consideration on the issues that I raised.<br><br>Opinions for the revised paper for my main concerns<br>1) About statistically validated surrogate endpoints: The change made on this concern is superfluous; the addition of a few sentences without any reference or serious discussions for statistical validation methods. I provided two important references for authors to explore the importance of statistical validations on surrogates and its clinical relevance. I regret to see they are simply ignored.<br>2) I simply replicate my concern again since I did not see any change.<br>3) There are not serious considerations for the issues I raised and about the reference I mentioned.	Thank you for reviewing our revised manuscript. We agree that whilst our project does not address statistical validation it is important to have a summary of statistical methods used. We have therefore added literature to this effect (11 references) including the two references that you suggested.<br><br>We provide a point-by-point response to comments from the first peer review, see below	The following statements have now been added in page 4, last paragraph:<br><br>*To be regarded as a valid surrogate endpoint, a biomarker or intermediate outcome is required: 1) to reliably predict the PRFO in individual trial participants ('individual level' or 'patient-level' surrogacy); and 2) the intervention effect on the surrogate endpoint should reliably predict the intervention effect on the PRFO ('trial-level' surrogacy) based on evidence from meta-analyses of RCT data on both outcomes[9 10]. Statistical surrogate validation uses various statistical methods, including meta-analyses of RCT aggregate and/or individual patient data[11 12], principal stratification [13], causal inference [14 15], bivariate network meta-analysis methods[16 17] and information theory [18]. However, surrogate validation should extend beyond statistical validity to include a multifaceted approach comprising of biological plausibility rationale and "face validity" of the surrogates in trials[19].*

For peer review only - http://bmjopen.bmj.com/site/about/guidelines.xhtml

**Comments from first peer review**		
The idea of the protocol is reasonable since many RCTs using surrogate endpoints appear to be ad-hoc. Some guidelines are certainly helpful to improve the relevance of the RCTs aiming at improving patients' benefit. Authors may consider three items when they revise the paper.<br><br>1. It is desirable for a surrogate endpoint to be validated statistically before it replaces the true endpoint. This validation process typically use the "correlation" between the true and surrogate endpoints. I refer the book of "The evaluation of surrogate endpoints", and [1] for this point. This important point is missing in the paper, and hence, I am afraid the use of uncorrelated surrogates in RCTs. While it may not be reasonable to impose statistically validated surrogate endpoints in all RCTs, some comments/considerations will be helpful in the process of developing the guideline. The statistical validation guarantees that a surrogate endpoint is correlated to the true endpoint at both trial-level and patient-level.<br><br>[1] Green, E. M., Yothers, G., & Sargent, D. J. (2008). Surrogate endpoint validation: statistical elegance versus clinical relevance. Statistical Methods in Medical Research, 17(5), 477-486.	Thank you, please see our response above.	We have now added 11 references on statistical validation methods including the reference you have provided, on last paragraph on page 5:<br><br>*To be regarded as a valid surrogate endpoint, a biomarker or intermediate outcome is required: 1) to reliably predict the PRFO in individual trial participants ('individual level' or 'patient-level' surrogacy); and 2) the intervention effect on the surrogate endpoint should reliably predict the intervention effect on the PRFO ('trial-level' surrogacy) based on evidence from meta-analyses of RCT data on both outcomes[9 10]. Statistical surrogate validation uses various statistical methods, including meta-analyses of RCT aggregate and/or individual patient data[11 12], principal stratification [13], causal inference [14 15], bivariate network meta-analysis methods[16 17] and information theory [18]. However, surrogate validation should extend beyond statistical validity to include a multifaceted approach comprising of biological plausibility rationale and "face validity" of the surrogates in trials[19].*
2. Introduction could be improved in different ways.<br>- The word "surrogate primary endpoint" is confusing to me and may be changed to "surrogate endpoint".<br>When one uses the term "surrogate endpoint", there	Thank you for this observation. Our response to this remains the same as earlier responded to: that the completed SPIRIT|CONSORT-SURROGATE guidelines will target trials	We have defined a surrogate primary endpoint on first use of the term: *surrogate primary endpoints (surrogate endpoint as the*

For peer review only - http://bmjopen.bmj.com/site/about/guidelines.xhtml

exists the "primary endpoint" or "true endpoint". Therefore, any surrogate endpoint cannot be the primary endpoint (at least in theory). Alternatively, the authors could use "surrogate endpoint as a primary endpoint". In addition, the definitions of "surrogate endpoints" and "the true endpoint" should be clearly defined without confusion. At least, I do not think "primary final patient relevant outcome" is a good word (P.4). It could be "true endpoint".	whose primary outcome is a surrogate endpoint and so we needed to be clear of that context. We have clarified this on the first time use of "surrogate primary endpoint".<br><br>We have defined surrogate endpoints as biomarkers and intermediate outcomes that substitute for and predict for a final patient/participant relevant outcome (i.e., characteristic or variable that captures how a patient feels, functions, or how long they survive, such as the outcomes of mortality or health-related quality of life) on page 5, second paragraph<br><br>We acknowledge that there isn't consensus on reference and definitions of these terms including "true endpoints" hence one of our research questions is to explore how surrogate endpoints are defined.	*primary outcome)* on the abstract on page 2 and introduction on page 4.<br><br>We have also added the following statement to rationalise focus of surrogate endpoints when used as primary outcomes on page 5: *The extensions focus on trials using surrogate endpoints as primary outcomes (including as part of a composite outcome) as these would inform trial conclusions and interpretations of results and possible approval of interventions*
3. There are a large number of surrogate endpoints, some of them are valid and others are invalid.<br>An example of valid surrogate endpoints is helpful for readers who are not familiar with the topic.<br>For instance, based on my knowledge, DFS (and PFS in advanced disease) are valid surrogates for OS in colorectal cancer [2].<br><br>[2] Buyse, M., Burzykowski, T., Michiels, S., & Carroll, K. (2008). Individual-and trial-level surrogacy in colorectal cancer. Statistical Methods in Medical Research, 17(5), 467-475.	Thank you for this suggestion. We have now clarified after the definition that reference to surrogate endpoints refers to both validated and non-validated surrogates that are reasonably likely to predict benefit and given examples of both citing:<br><br>Fleming TR, Powers JH. Biomarkers and surrogate endpoints in clinical trials. Stat Med. 2012 Nov 10;31(25):2973-84; Buyse M, Burzykowski T, Michiels S, et al. Individual- and trial-level surrogacy in colorectal cancer.	We have added the following on page 5-6, second paragraph:<br><br>*Additionally, reference of surrogate endpoints in this project refers to statistically validated surrogate endpoints (e.g., change in systolic blood pressure for cardiovascular mortality in anti-hypertensive treatments[29 30], disease-free survival (and progression free survival in advanced disease) in colorectal cancer[31]) and non-validated surrogates for which are 'reasonably likely to predict health*

For peer review only - http://bmjopen.bmj.com/site/about/guidelines.xhtml

	Stat Methods Med Res 2008;17(5):467-75; FDA. Table of Surrogate Endpoints That Were the Basis of Drug Approval or Licensure 2022; FDA-NIH Biomarker Working Group. BEST (Biomarkers, endpoints, and other tools) resource	*benefit' (e.g., reduction in amyloid load in Alzheimer's disease)*[29 32]

For peer review only

For peer review only - http://bmjopen.bmj.com/site/about/guidelines.xhtml
